# Influence of the permittivity between fillers and binders on the properties of upside-down composites for recycling purposes

**DOI:** 10.1039/d5ma00554j

**Published:** 2025-07-28

**Authors:** Sivagnana Sundaram Anandakrishnan, Mikko Nelo, Mohadeseh Tabeshfar, Viktoria Kraft, Neamul Hayet Khansur, Jani Peräntie, Yang Bai

**Affiliations:** a Microelectronics Research Unit, Faculty of Information Technology and Electrical Engineering, University of Oulu FI-90570 Oulu Finland yang.bai@oulu.fi; b Infotech Oulu FI-90570 Oulu Finland; c Department of Materials Science and Engineering, Friedrich-Alexander-Universität Erlangen-Nürnberg (FAU) 91058 Erlangen Germany; d Department of Materials Science and Engineering, Case Western Reserve University Cleveland OH 44106 USA

## Abstract

Upside-down composites have recently advanced towards recycling piezoceramics. However, the recycled piezoceramics retain only about 10–30% of the pristine piezoelectric properties. To date, there has been no systematic study on the origin of this limitation and on potential routes for improving these recycled materials. This work addresses this issue by combining empirical and modelling evidence. The phenomenon of the influence of disparate permittivity between the fillers and binders is explained by fitting experimental parameters from sets of lead-based and lead-free upside-down composite samples into the Lichtenecker and Yamada models. Results suggest that for high filler contents, the biasing field permeation caused by the binders that leads to lower piezoelectric properties can be experimentally confirmed and correctly modelled. For lower filler contents, the models significantly deviate from the experimental data due to the distinctive shaping method. This issue indicates the necessity of developing a new theoretical methodology for upside-down composites.

## Abstract 2

The theoretical role of filler permittivity in the properties of upside-down composites with high filler content is experimentally validated, promoting the upscaling of recycled piezoelectric ceramics for a second life in sensing devices.

## Graphical abstract

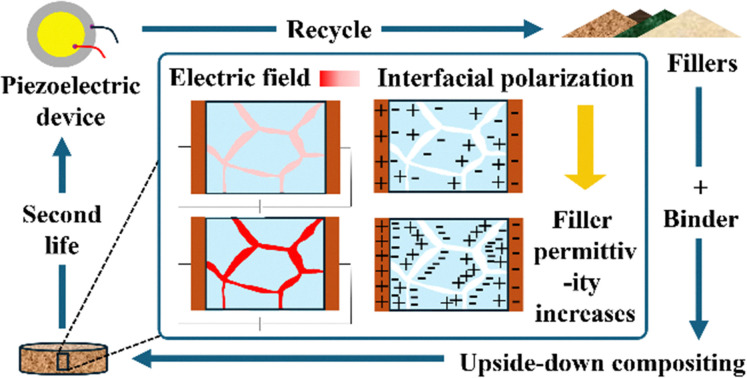

## 1. Introduction

Bulk piezoelectric ceramics or simply piezoceramics are a class of piezoelectric materials having an extensive amount of available compositions to choose from, each offering flexible functional tunability and facile formability for various device requirements.^1^ Typically, the production of piezoceramics utilizes the solid-state route, which involves a phase formation step known as calcination, followed by a grain-growth and densification step known as sintering. The criticality of the sintering step is emphasized given that the extent of densification and the grain size in the piezoceramics are essential for obtaining the desired properties.^1,2^ However, sintering is typically carried out at temperatures higher than 1000 °C, demanding a large energy budget to produce the desired piezoelectric functionality. The large energy budget eventually leads to a large ecological and carbon footprint in the piezoelectric industry.^3^

Researchers have investigated several possible approaches to reduce the energy budget of manufacturing piezoceramics. The investigations range from the material design stage (*e.g.*, simulating functional properties using first-principles density functional theory, molecular dynamics, phenomenological phase field models, and machine learning models), the manufacturing stage (*e.g.*, sintering with sintering aids, cold sintering, low- and ultra-low-temperature co-firing, and room-temperature densification), and the deployment stage (*e.g.*, piezoelectric energy harvesting and circuitry development for self-sufficient electronics) to the post-deployment or recycling stage (*e.g.*, upside-down composite method).^3^

Recently, recycling old, retired, worn-out, or rejected piezoceramics has been explored as a viable solution to reduce the energy budget for producing materials with a high piezoelectric voltage coefficient (*g*), utilizing the upside-down composite method.^4,5^ The energy budget for the production of second-life piezoceramics *via* this recycling method is observed to be two-orders of magnitude smaller than that for sintering new piezoceramics.^4^ The recycled materials are still able to surpass their pristine counterparts in terms of sensing capability (*i.e.*, high *g* values). Both the advantages of energy consumption and the performance motivated us to study the behaviour of these recycled materials deeper.

In this recycling method, a suitable binder phase is mixed with the crushed piezoceramic filler phase, where the filler and binder form a core–shell microstructure.^6^ Upon the application of high pressures with or without mildly elevated temperatures, the binder phase reprecipitates at sites away from the contacting filler surfaces, driving densification.^7^ The resulting material is a ceramic-based composite termed an upside-down composite owing to the high filler fractions (>75 vol%) that can be achieved compared to those of ordinary composites (up to 60 vol%).^8–14^

The large *g* values commonly seen in upside-down composites are attributed to their low permittivity arising from the dominance of binder on electric field.^15^ The cause behind this effect is the disparate permittivity values between the filler and binder, which consequently implies significantly suppressed charge mobility in the binder.^16^ An analogous phenomenon is also widely seen in porous piezoceramics.^17–20^ Although a low composite permittivity is beneficial for producing large *g* values, the lack of charge mobility in the binder increases the charge relaxation time in the composite, leading to inefficient poling, and consequently a piezoelectric charge coefficient (*d*) an order of magnitude lower than that of piezoceramic counterparts.^3,4,21^

Moreover, inefficient poling also occurs because of the large regional permittivity mismatch forcing the electric field to follow a non-linear and disrupted path,^22^ which further leads to a wider distribution of the electric field with lower magnitudes in the vicinity of the active filler phase. This ultimately increases the coercive field of the composite, which even possibly exceeds its breakdown strength.^19^ In this case, a complete poling state may never be achieved before the material fails.

Restricted by this fundamental issue, further optimizing the piezoelectric properties of upside-down composites may be challenging without being able to systematically browse through the available filler and binder options, and thus select the most promising ones. This would be a tedious procedure under the currently used, solely experimental approaches. Meanwhile, to tune the composite properties for different specific applications, it is imperative to predict the behavior of materials given their respective constituents as well as other relevant parameters, and more importantly to simulate the effect of the dominance of binder on electric field, as described previously.^22^ All these potential developments require the involvement of theoretical supports, as is the case for ordinary composites.

In the literature, a number of theoretical models have been reported for the purpose of predicting the permittivity and piezoelectric coefficients of composites based on a series of necessary input parameters, boundary conditions, and phase inter-connectivity types.^23,24^ Promising results have been achieved with regards to fitting the experimental data to models.^16,25–28^ Recently, a modified Lichtenecker model has been successfully applied to upside-down composites with varying filler volume fractions, whilst considering the contribution from their porosity.^29^ Nevertheless, collaborative experiment-modelling approaches with controlled input parameters are still lacking, although these approaches are essential for the future-oriented machine learning-based assistive methods for designing new upside-down composites, especially for the high filler content that is the key for success in the recycling of piezoceramics.

Therefore, in this work, a systematic investigation was carried out through a combination of experiments and modelling to reveal the contribution of the filler permittivity to the global permittivity as well as the *d* of upside-down composites. It is worth noting that in this study, the fillers are carefully chosen so that within each set of fillers, the *d* values were controlled to be identical, but the permittivity values vary substantially. This intentional experimental control of the input parameters to the models was performed to highlight and directly validate the universal effect of biasing of the supplied electric field in the low permittivity binder, and thus develop the method towards the prediction of the functionality of recycled piezoceramics.

Through a combined experiment-modelling approach, this work validates that the existing discrepancies between the models and experimental data are attributed to the fact that the applicability of a model to fit the dielectric and piezoelectric data of composites is based on the assumed boundary conditions used to derive the model, which will change with the fabrication technique used.

## 2. Materials and methods

### 2.1 Fabrication of piezoceramic pellets and fillers

Table 1 summarizes the specimens fabricated in this work, including information on the filler compositions, binder volume fractions (where applicable), and densities. Each type of specimen is assigned a sample ID and the results for each sample with the same ID were obtained by averaging at least three specimens. A total of six distinct ceramics based on three Pb-based (sample ID prefix: PT) and three Pb-free (sample ID prefix: BT) compositions were fabricated using the solid-state route. All ceramic powders were synthesized in the laboratory except for PT 1 (PZ29) and PT 2 (APC-855), which were purchased commercially from Meggitt A/S (Denmark) and American Piezo International (APC) Ltd (USA), respectively.

**Table 1:** Summary of the compositions and densities of the specimens studied in this work

Sample ID	Form of material	Filler composition	Binder volume fraction (%)	Measured density (g cm^−3^)	Theoretical density (g cm^−3^)	Relative density (%)
PT 1-P	PZ29 ceramic	—	—	∼7.46	7.91 ± 0.10	94.3 ± 1.2
PT 2-P	APC-855 ceramic	—	—	∼7.57	7.73 ± 0.10	98.0 ± 1.2
PT 3-P	0.67Pb(Mg_1/3_Nb_2/3_)O_3_–0.33PbTiO_3_ ceramic	—	—	∼7.59	8.10 ± 0.07	93.7 ± 0.8
BT 1-P	(Ba_0.80±0.02_Ca_0.14±0.005_)(Ti_0.90±0.005_Zr_0.10±0.005_)O_2.94±0.02_ ceramic	—	—	∼5.02	5.47 ± 0.06	91.7 ± 1.1
BT 2-P	(Ba_0.95±0.01_)(Ti_0.94±0.005_Sn_0.06±0.005_)O_2.95±0.01_ ceramic	—	—	∼5.82	5.90 ± 0.04	98.6 ± 0.6
BT 3-P	(Ba_0.79±0.01_Ca_0.14±0.005_)(Ti_0.88±0.005_Zr_0.12±0.005_)O_2.94±0.02_ ceramic	—	—	∼5.06	5.50 ± 0.06	92.1 ± 1.0
PT 1-C	Upside-down composite	PT 1	∼18.8	5.86 ± 0.03	∼6.76	86.6 ± 0.5
PT 2-C	Upside-down composite	PT 2	∼18.4	6.01 ± 0.05	∼6.64	90.5 ± 0.8
PT 3-C	Upside-down composite	PT 3	∼19.1	6.01 ± 0.04	∼6.89	87.1 ± 0.5
BT 1-C	Upside-down composite	BT 1	∼13.8	4.65 ± 0.04	∼4.97	93.7 ± 0.8
BT 2-C	Upside-down composite	BT 2	∼14.7	5.01 ± 0.04	∼5.30	94.6 ± 0.7
BT 3-C	Upside-down composite	BT 3	∼13.8	4.77 ± 0.04	∼4.99	95.6 ± 0.8

Reactants of PbO (99.9%, Sigma-Aldrich, USA; 99.9%, Thermo Scientific, USA), MgO (≥99%, Sigma-Aldrich, USA), Nb_2_O_5_ (99.9%, Sigma-Aldrich, USA) and TiO_2_ (99.8%, Alfa Aesar, USA) were used for 0.67Pb(Mg_1/3_Nb_2/3_)O_3_–0.33PbTiO_3_ (PT 3); BaZrO_3_ precursors were synthesized from BaCO_3_ (99%, Thermo Scientific, Germany) and ZrO_2_ (99.6%, Aldrich Chemistry, USA) for (Ba_0.80±0.02_Ca_0.14±0.005_)(Ti_0.90±0.005_Zr_0.10±0.005_)O_2.94±0.02_ (BT 1), and from BaCO_3_ (99%, Alfa Aesar, Germany) and ZrO_2_ (99% Sigma Aldrich, UK) for (Ba_0.79±0.01_Ca_0.14±0.005_)(Ti_0.88±0.005_Zr_0.12±0.005_)O_2.94±0.02_ (BT 3).^30^ Subsequently, the rest of the appropriate reactants were mixed to synthesize (1) BT 1 from BaCO_3_ (99%, Thermo Scientific, Germany), CaCO_3_ (99%, Sigma Aldrich, USA), TiO_2_ (99.8%, Aldrich Chemistry, Canada) and its precursor, (2) (Ba_0.95±0.01_)(Ti_0.94±0.005_Sn_0.06±0.005_)O_2.95±0.01_ (BT 2) from BaCO_3_ (99.8%, Thermo Fisher Scientific, USA), SnO_2_ (99.9%, Thermo Fisher Scientific, USA) and TiO_2_ (99.6%, Thermo Fisher Scientific, USA), and (3) BT 3 from BaCO_3_ (99%, Alfa Aesar, Germany), CaCO_3_ (99%, Sigma Aldrich, USA), TiO_2_ (99.8%, Aldrich Chemistry, Canada) and its precursor.

Firstly, the reactants were weighed accurately according to their stoichiometries, and subsequently mixed on a planetary ball mill. It should be noted that before mixing, the reactants for BT 1 as well as those for its precursor were individually milled with ZrO_2_ beads at 150 rpm for 12 h in ethanol, and then sieved through a 200 μm mesh after drying as an extra control step. The other reactants were used in their pristine form. The medium was ethanol and the mixing and milling conditions were as follows: (1) with ZrO_2_ beads at 150 rpm for 6 h for PT 3, (2) without any beads at 100 rpm for 6 h for BT 1 and its precursor, (3) with ZrO_2_ beads at 70 rpm for 24 h for BT 2, and (4) with ZrO_2_ beads at 100 rpm for 6 h for BT 3 and its precursor.

The mixtures were placed in Al_2_O_3_ crucibles and calcined at 700 °C for 4 h for PT 3, at 1400 °C for 4 h for BT 1 and its precursor, at 1200 °C for 6 h for BT 2, at 1200 °C for 4 h for the precursor of BT 3, or at 1150 °C for 4 h for BT 3. The as-purchased PT 1 and PT 2 ceramic powders, as well as the calcined powders, were ball-milled again with ZrO_2_ beads in ethanol at 150 rpm for 12 h (PT 1, PT 2, PT 3, BT 1 and BT 3) or at 70 rpm for 72 h (BT 2). The dried powders were sieved through a 200 μm (PT 1, PT 2, PT 3, BT 1 and BT 3) or 100 μm (BT 2) mesh, and then shaped into green bodies with a diameter of 10 mm under a uniaxial pressure of 90–110 MPa (PT 1, PT 2, BT 1, BT 2 and BT 3) or 40 MPa (PT 3). Polyvinyl alcohol was used as the binder, which was burnt at 550 °C. The green bodies were sintered in Al_2_O_3_ crucibles at 1150 °C for 2 h (PT 1 and PT 2) or at 1200 °C for 4 h (PT 3), or on Pt substrates at 1400 °C (BT 1 and BT 3) or at 1380 °C (BT 2) for 4 h. Powder beds of the same corresponding compositions were utilized for the Pb-based samples to help suppress Pb loss.^1^

A part of the sintered ceramics was structurally and electrically characterized, while the rest was crushed with a hydraulic press. Mesh sizes of 425 μm, 180 μm, and 63 μm were utilized to select particles with a size in the range of 63–180 μm. These particles were employed as the fillers for the fabrication of the composites.

### 2.2. Synthesis of binder

An organometal halide perovskite compound, (PTMA)CdCl_3_ ^4^ where PTMA is C_6_H_5_N(CH_3_)_3_, was synthesized in-house to be used as the binder in the composites. (PTMA)Cl (≥98%, Sigma-Aldrich, USA) and CdCl_2_ (99.99%, Sigma-Aldrich, USA) as the reactants were weighed stoichiometrically and a solution of acetonitrile in deionized water with a volume fraction of 60% was used to dissolve the reactants in a concentration of 13.3 w/v%, forming a solution containing C_6_H_5_N(CH_3_)_3_^+^, Cd^2+^ and Cl^−^. Subsequently, this solution was mixed using a magnet stirrer for 24 h^31^ and passed through a 0.2 μm polytetrafluoroethylene filter. (PTMA)CdCl_3_ crystals were grown *via* slow static precipitation for 3–4 weeks.

### 2.3. Fabrication of upside-down composites

A suspension was first prepared by mixing 60 w/v% of fillers in 0.6 v/w% (PTMA)CdCl_3_ acetonitrile-deionized water solution. Coated fillers were obtained after evaporating the solvents.^5^ Then, the coated fillers were mixed homogenously with 5 wt% crushed (PTMA)CdCl_3_ crystals with a mortar and pestle. This binder concentration was chosen *via* extensive experimental trials, where it was found that 5 wt% was the minimum required binder content for the composite to be intact, while optimizing its properties.^5^ 3–4 drops of a transport phase consisting of the saturated (PTMA)CdCl_3_ solution that was utilized for the synthesis of (PTMA)CdCl_3_ was added for every gram of filler. Subsequently, the filler-binder mixture was loaded into a cylindrical die made from hardened steel with an inner diameter of 10 mm, followed by hot pressing at 150 °C and 250 MPa for 30 min. The apparatus was cooled down to 40 °C, whilst maintaining the pressure before the sample was demolded. The amount of filler used to prepare each sample is listed in Table S1 in the SI. Pellets of pure crushed (PTMA)CdCl_3_ crystals were also made using the identical fabrication procedure as that for the composites but without the addition of the fillers.

### 2.4. Structural and electrical characterization

The density of the samples was calculated based on their measured weight divided by volume. XRD (X-ray diffractometry, AXS D8 Discover, Bruker, Germany) was carried out under Cu Kα radiation (*λ*_kα1_ = 1.5406 Å, *λ*_kα2_ = 1.54439 Å, and wavelength ratio = 0.5) at a 2*θ* scanning rate of 0.02° min^−1^ to identify the phases. Rietveld refinement was carried out using the SmartLab Studio-II software. EPMA (electron probe microanalysis, JXA-8530F Plus, JEOL, Japan) with a 15 kV acceleration voltage, 15 nA probe current, and 1–5 μm beam spot size was used for elemental analysis. FESEM (field emission scanning electron microscopy, ULTRA Plus, Carl Zeiss SMT AG, Germany) equipped with EDS (energy dispersive X-ray spectroscopy) operating at an acceleration voltage of 15 kV was used to examine the microstructure. The ImageJ software was used to obtain the volume fraction of each phase in the composites from the FESEM micrographs based on three different surfaces and six probing locations within each surface. Ag-epoxy paste (H20E-4GM, Epoxy Technology, USA) cured at 150 °C for 30 min and Ag conductor paste (5065, Dupont, UK) cured at 130 °C for 10 min were coated on the ceramic and composite surfaces, respectively, as the electrodes.

The samples were processed to different levels of finish for different purposes. The ceramic surfaces were polished on silicon carbide abrasive papers with grit sizes of P1200 and P2500 before coating the electrodes. In the case of EPMA, the ceramic surfaces were further polished on P4000 abrasive paper, and then with a diamond suspension with a particle size of 1 μm (Struers, France). For FESEM, the cross-sectional surfaces of the composites were not only subjected to polishing on P1200, P2500 and P4000 abrasive papers, consecutively, but also an additional ion polishing performed under vacuum inside a cooling cross-section ion polisher (IB-19520CCP, JEOL, Japan) with a stainless-steel source. The surfaces of the sample for EPMA and FESEM were coated with carbon.

The theoretical density (*ρ*_f_) of each filler material was calculated using eqn (1), where *N*_A_ is Avogadro's number, *N* is the number of atoms per unit cell, *A* is the molecular weight of the nominal chemical formula calculated from the EPMA results, and *V* is the unit cell volume obtained from the XRD results. The binder volume fraction (*φ*_b_) in the composite was calculated using eqn (2), where *ρ*_b_ is the theoretical density of (PTMA)CdCl_3_ (∼1.80 g cm^−3^)^31^ and *m*_f_ is the mass fraction of filler (0.95 for all samples). The theoretical density of the composite (*ρ*_c_) was calculated using eqn (3), where *φ*_f_ is the filler volume fraction.1
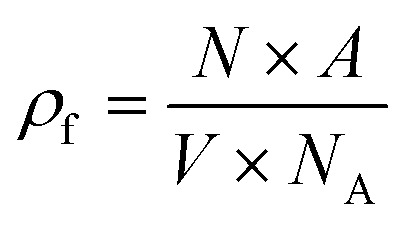
2
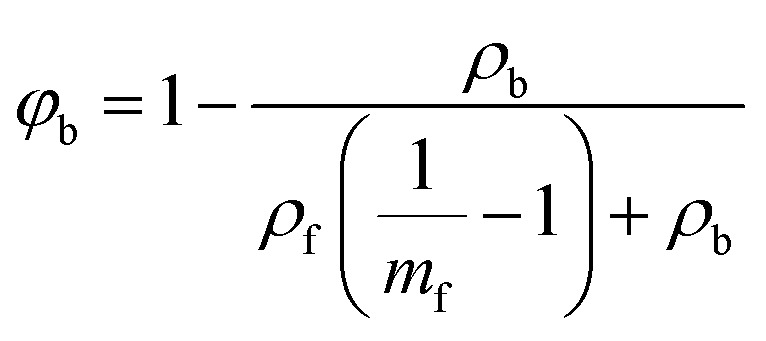

3 *ρ*_c_ = *ρ*_b_*φ*_b_ + *ρ*_f_*φ*_f_

The samples with electrodes were measured for their dielectric and piezoelectric properties using a combination of LCR meter (E4980AL, Keysight Technologies, USA), Berlincourt *d*_33_ meter (YE2730A, APC International Ltd, USA) under 0.25 N alternating force at 110 Hz, and impedance analyzer (E4990A, Keysight, USA). The conductivity of the samples was measured using a source meter (Model 2450, Keithley, USA). The poling was performed at 3 kV mm^−1^ for 10 min in a silicone oil bath and the poled samples were electrically shorted for 24 h before measurements. The poling and measurements were both conducted at room temperature and in the dark.

## 3. Results and discussion

Data associated with this work are openly available, see ref. 32.

### 3.1. Specimens and models

The resulting samples are ceramic–ceramic composites (oxide perovskite ceramic filler and crushed halide perovskite crystal binder) with a 0–3 (filler-binder) connectivity. It should be first emphasized that the design of this work is to find fillers with the same *d*_33_ values but with vastly different permittivity without a significant change in chemical composition. This was not an easy task as it is widely known that usually for the same compositional family, the *d*_33_ and permittivity evolve simultaneously in the same directions. After extensive experimental trials, two sets of fillers were chosen for this study. One set was Pb-based, consisting of two donor-doped soft-type Pb(Ti,Zr)O_3_ (PZT),^33^ and 67Pb(Mg_1/3_Nb_2/3_)O_3_–33PbTiO_3_.^34^ All three fillers show the same *d*_33_ values of about 490 pC N^−1^. The other set is Pb-free compounds, which comply with the EU's RoHS (Restriction of Hazardous Substances in Electrical and Electronic Equipment) directive.^35,36^ They contain two variants from (Ba,Ca)(Zr,Ti)O_3_ (BCZT)^37^ and Ba(Sn,Ti)O_3_ (BST),^38^ all showing the same *d*_33_ values of about 330 pC N^−1^.

However, the results of this work should never be interpreted by attempting to compare the Pb-based and Pb-free samples due to their simultaneously varying *d*_33_ and permittivity values. One can only compare within each set, where the *d*_33_ is kept the same with varying permittivity. In addition, the selection of the fillers in this work was solely for the sake of searching for identical *d*_33_ but different permittivity within the same compositional family, rather than by simply grouping according to whether containing Pb or not. The additional benefit here is that in our previous study, the PbTiO_3_-based and BaTiO_3_-based fillers were demonstrated to be feasible for the recycling procedure owing to the large values of *g*_33_ obtained in their composites, giving the recycled materials a second life for sensor applications.^4^ Moreover, these two types of compounds are widely used in many electronic devices, which can be potential sources of recycled materials.^39–41^

Both the (PTMA)CdCl_3_ crystal alone and the crushed (PTMA)CdCl_3_ crystals as a binder in upside-down composites have been thoroughly researched in terms of chemical stability, dielectric constant, phase and microstructure, aging, and functional properties.^4,5,31^ This fact provides the basis for choosing (PTMA)CdCl_3_ as the binder for a more fundamental study on upside-down composites in this work. Although the toxic nature of this Cd-containing compound can be a concern, this study used this binder only for proof-of-concept purposes owing to its success in previous upside-down composites.^4,5^ Non-toxic binders, such as Mn-based halide perovskites,^42^ are available and can be investigated for recycling in the future. Moreover, the amount of binder used is minimal (only 5 wt%) and much lower than that of the filler, which may contain a much larger amount of toxic Pb. Based on this aspect, using the minimal Cd-containing binder to recycle Pb-containing fillers is less likely to cause a real problem.

As can be seen in Table 1, this work studied six types of oxide perovskite-structured piezoceramics, PT 1-P, PT 2-P, PT 3-P, BT 1-P, BT 2-P, and BT 3-P. Correspondingly, six types of composite samples were also studied. They are PT 1-C, PT 2-C, PT 3-C, BT 1-C, BT 2-C, and BT 3-C, which were fabricated using the above-mentioned six types of ceramics as the fillers, respectively, and the binder. The dimensions and pictures of the samples fabricated in this work are displayed in Table S1 and Fig. S1, respectively, in the SI. Note that the ceramics and composites were fabricated with comparable dimensions (*i.e.*, diameter of ≈10 mm and thickness of 1–2.5 mm) to ensure a valid comparison of the dielectric and piezoelectric properties among them.

The measured permittivity and *d* values of the resulting composites were compared with the values obtained using the Lichtenecker model^43^ and the Yamada model,^24^ as given in eqn (4) and (5), respectively.

4 *ε*^*k*^_c_ = *ε*^*k*^_f_*φ*_f_ + *ε*^*k*^_b_*φ*_b_

5
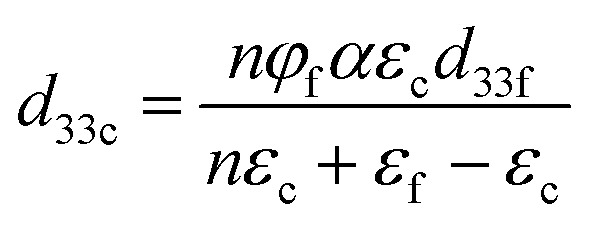
In eqn (4) and (5), subscripts c, b, and f refer to the composite, the binder phase, and the filler phase, respectively. In practice, the electrical properties of the filler can be represented by the properties of the corresponding ceramic fabricated from the same material, since each filler particle was in fact a small piece of the same ceramic. *ε* corresponds to the relative permittivity, *φ* corresponds to the volume fraction of a phase in the composite, *d*_33_ refers to the longitudinal piezoelectric charge coefficient, *n* refers to a constant, which is the inverse of the depolarization factor describing the shape anisotropy effects of the fillers, *α* refers to a constant that quantifies the poling efficiency of the composite, and *k* refers to a constant describing the specific microgeometry of the fillers in the composite.

### 3.2. Phases of fillers and microstructure of composites

**Fig. 1:**
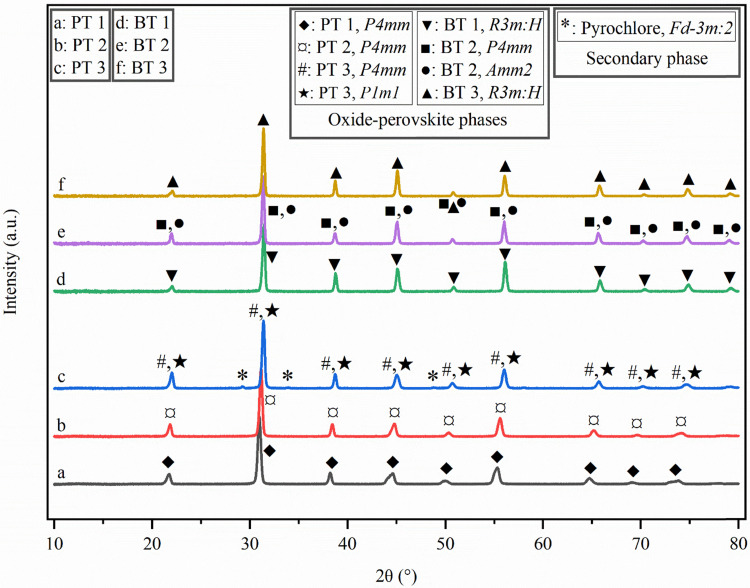
X-ray diffractograms of all the piezoceramic fillers fabricated in this work: (a) PT 1, (b) PT 2, (c) PT 3, (d) BT 1, (e) BT 2, and (f) BT3.

Fig. 1 shows the X-ray diffractograms of the fillers with the identified space groups marked for the major oxide perovskite phases and the minor secondary phases. The major oxide perovskite phases matched well to the known phases, indicating complete and expected phase formation. The Rietveld refinement results along with the refinement parameters, as shown in Fig. S2 in the SI, assigned the phases with good fits to the diffractogram of each filler, which gave favorable *R*_p_ values (<6%). Details of the refinement including the corresponding peak lists and information on the assigned phases are provided in Tables S2–S13 in the SI.

According to the refinement results, the space group of *P*4*mm* (tetragonal perovskite phase) matched PT 1 (PDF card 01-070-6380), PT 2 (PDF card 04-023-9159), and PT 3 (PDF card 04-026-8676). Pure, single perovskite phases were formed in PT 1 and PT 2 without signs of secondary phases. However, another perovskite phase (*P*1*m*1, monoclinic, PDF card 04-024-5195) matched PT 3 simultaneously, indicating that PT 3 was in its morphotropic phase boundary (MPB) at room temperature, in line with the chemical composition calculated from the EPMA results shown in Table S14 in the SI.^44,45^ A secondary phase was also present in PT 3, where the reflections at 2*θ* of 29.22°, 33.88°, 48.81°, and 57.97° matched with a cubic pyrochlore phase (space group *Fd*3̄*m* choice 2, PDF card 01-084-1731), which is commonly seen in the literature.^46^

The space group of *R*3*m*:*H* (rhombohedral perovskite phases) matched BT 1 and BT 3 (PDF card 04-025-4917), while BT 2 matched the space groups of *P*4*mm* (PDF card 04-007-5135) and *Amm*2 (orthorhombic perovskite phase, PDF card 01-085-9628). No secondary phase was observed in these three Pb-free, BT-based fillers. The EPMA results in Table S14 also explain the co-existence of the two perovskite phases in BT 2-P, as it was expected to be at its MPB.^38,47^

**Fig. 2:**
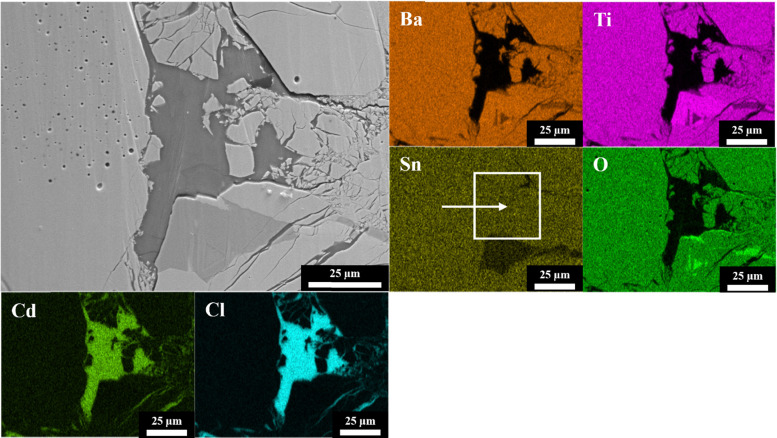
FESEM micrograph and EDS maps of the BT 2-C sample.

Fig. 2 shows the cross-sectional FESEM image of an upside-down composite sample, BT 2-C, along with its corresponding EDS maps. Clear boundaries between the filler and binder, indicated by the distinctive regions containing Ba/Ti/O and Cd/Cl, were evident. However, in addition to the filler, Sn was also seen to cover a part of the binder area (see the white box marked with a white arrow in the EDS map of Sn in Fig. 2). This is a detection error and was attributed to the overlap of the X-ray L_α_ signals between Sn (L_α_ = 3.443 keV) in the filler and Cd (L_α_ = 3.133 keV) in the binder, and this issue is not uncommon with the EDS technique.^48^ The resolution depth of the EDS probe was approximately 1–2 μm, meaning that the X-ray was likely to penetrate the very thin binder layers, and thus reach the filler underneath. Meanwhile, no overlap of the Cd signal was witnessed in the filler regions due to the larger quantity of fillers, which diminished any possible Cd signal.

Nevertheless, according to Fig. 2 as well as Fig. S3–S7 in the SI, which show the cross-sectional FESEM images and EDS maps of the other composite samples (PT 1-C, PT 2-C, PT 3-C, BT 1-C, and BT 3-C), the expected high-quality microstructure, which successfully replicated its upside-down composite counterparts, was obtained in all the composites in this work, owing to the optimized preparation methodology with highly dense ceramic fillers (>92%, see Table 1) and (PTMA)CdCl_3_ as the binder.^4,5^ No prominent voids or cracks were noticed within the filler and binder phases and between their interfaces. The high filler volume fraction could also be visualized in the FESEM images, while no cross-reactions between the fillers and binder were observed according to the EDS maps.

As an additional step to quantify the volume fractions of each phase obtained in the composites, Table S15 confirms that the average area fraction of the binder phase was 19.2% ± 0.8% for sample PT 2-C, matching the designed value of 18.4 vol% (with an error of <0.05, which is within the accuracy range of the electronic balance used to weigh the compounds). According to this observation, it can also be concluded that the binder volume fractions within each set of samples (Table 1) can be considered identical, and hence the filler permittivity becomes the only variable for the composites.

### 3.3. Effect of the type of filler on dielectric and piezoelectric properties of the composites

A basic picture of the dielectric properties of the ceramics and composites studied in this work, represented by the evolution of their relative permittivity (*ε*_r_) and dielectric loss (tan *δ*) over the frequency range of 20 Hz to 100 kHz in the unpoled and poled states, is reflected by Fig. S8–S13 in the SI. It is evident that all the ceramic samples showed a decreasing trend in their *ε*_r_ and tan *δ* values with an increase in frequency but to different extents, owing to the interplay between contributions from the resonant frequencies of dipoles and those from the ferroelectric domains.^49^

The ceramic samples had poled permittivity values higher than their unpoled counterparts when possessing a tetragonal crystal structure (*i.e.*, PT 1-P and PT 2-P in Fig. S8a and S9a, respectively), whereas for the other crystal structures, the opposite trend was observed (*i.e.*, BT 1-P and BT 3-P in Fig. S11a and S13a, respectively). This is attributed to the dominance of the 180° domains in the tetragonal crystal structure and non-180° domains in the other crystal structures, respectively, after poling. For instance, in the tetragonal crystal structures, a larger number of populous 180° domains are reoriented in the direction of the applied field as opposed to rhombohedral crystal structures with pronounced reorientation of the 71° and 109° domains, away from the field. Both observations are consistent with the previous work.^4^ In the cases of PT 3-P and BT 2-P, their poled permittivity values were approximately equal to the unpoled values considering acceptable deviations (≈10%). As discussed above, PT 3-P and BT 2-P possessed compositions in their MPBs, where the coexistence of a tetragonal crystal structure and another crystal structure was observed. The presence of both types of crystal structures balanced the competing effects of the 180° and non-180° domains.

Similarly, the permittivity evolution with frequency for all the composite samples followed the same trend as that of the ceramics. The interfacial/space-charge polarizations between the filler and binder brought an extra factor that influences the change in permittivity.^50^ The poled permittivity values were always higher than their unpoled counterparts for all the composite samples. This phenomenon distinguishes that of the ceramics, owing to the positive ferroelastic contribution from the (PTMA)CdCl_3_ binder, which could ease the domain pinning effect on the fillers during poling.^4,31^ The detailed reasons for the contributions by the microstructure of the composite toward its dielectric properties are discussed in Section 5S in the SI.

The existence of space charge in the composites can be confirmed by analyzing the occurrences of relaxations in their dielectric spectra. Typically, they are observed at frequencies in the range of 100 kHz–10 MHz.^51,52^ Fig. S14a shows the dielectric spectra measured with the impedance analyzer for PT 2-C in its poled state in the corresponding frequency range. Five resonance-type dielectric dispersions are observed (marked with ①, ②, ③, ④, and ⑤ at 150, 400, 587, 787, and 900 kHz, respectively), each being characterized by a resonance peak and an anti-resonance trough in the permittivity and a peak in tan *δ* corresponding to the trough. These are related to the piezoelectric resonance of the oriented domains after poling and are typically observed in the literature.^4,51^

Beside the resonance-type dispersions, two relaxation-type dispersions (marked with ⑥ and ⑦ at 2.63 and 4.62 MHz, respectively) are also noticeable. Fig. S14b then compares the impedance (*Z*) and phase angle (*Θ*) of the same sample at the same frequencies. The resonance-type dispersions are further confirmed to be attributed to the resonance of the piezoelectric element owing to the resonance and antiresonance in *Z* and the corresponding peaks of *Θ*.^53^ In comparison, the relaxation-type dispersions possess no characteristic peaks or troughs in *Z* in Fig. S14b, implying that these peaks are related to the space charge between different phases in the composite.^54^

**Fig. 3:**
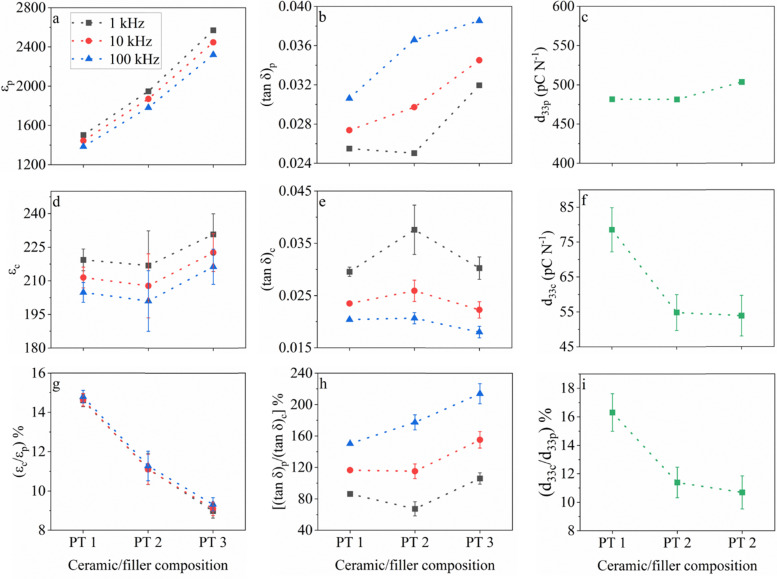
Variation in (a) ceramic relative permittivity (*ε*_p_), (b) ceramic dielectric loss ((tan *δ*)_p_), (c) ceramic *d*_33_ (*d*_33p_), (d) composite relative permittivity (*ε*_c_), (e) composite dielectric loss ((tan *δ*)_c_), (f) composite *d*_33_ (*d*_33c_), and ratio of (g) composite permittivity to ceramic permittivity ((*ε*_c_/*ε*_p_)%), (h) ceramic dielectric loss to composite dielectric loss (((tan *δ*)_p_/(tan *δ*)_c_)%) and (i) composite *d*_33_ to ceramic *d*_33_ ((*d*_33c_/*d*_33p_)%) shown as percentages for the Pb-based samples. The dielectric properties are extracted at frequencies of 1 kHz, 10 kHz and 100 kHz from the unpoled state. The dotted lines connecting the symbols are for visual convenience only and do not represent the evolution of the values between the datapoints.

**Fig. 4:**
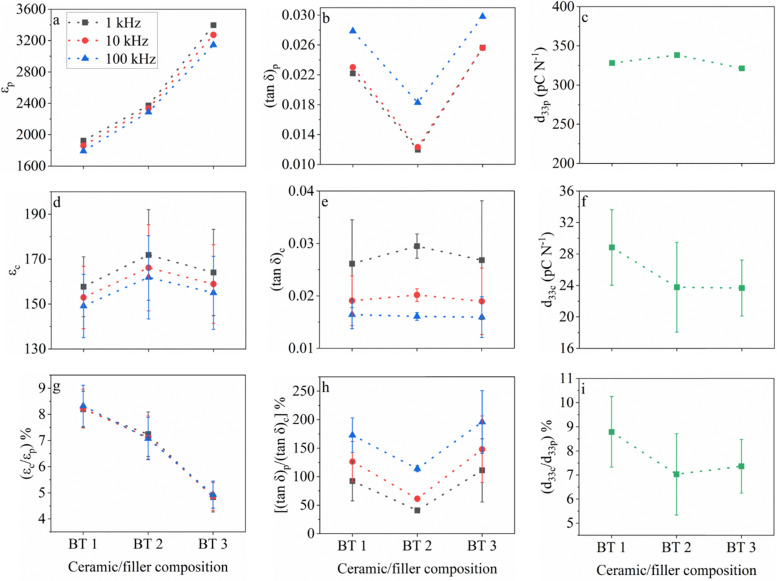
Variation in (a) *ε*_p_, (b) (tan *δ*)_p_, (c) *d*_33p_, (d) *ε*_c_, (e) (tan *δ*)_c_, (f) *d*_33c_, (g) (*ε*_c_/*ε*_p_)%, (h) ((tan *δ*)_p_/(tan *δ*)_c_)%, and (i) (*d*_33c_/*d*_33p_)% for the Pb-free samples. The dielectric properties are extracted at frequencies of 1 kHz, 10 kHz and 100 kHz from the unpoled state. The dotted lines connecting the symbols are for visual convenience only and do not represent the evolution of the values between the datapoints.

Fig. 3 and 4 show a comparison of the unpoled *ε*_r_ and tan *δ* values for the Pb-based and Pb-free sets of samples, respectively, obtained at frequencies of 1 kHz, 10 kHz and 100 kHz, along with the *d*_33_ values after poling between the ceramics and the corresponding composites. The percentages of these *ε*_r_, tan *δ*, and *d*_33_ values of the composites (subscript c) out of the values of the corresponding ceramic counterparts (subscript p) are also shown as an indication for the extent of property retention after being transformed from ceramics into upside-down composites.

The original design for the fillers is well reflected in Fig. 3a and c, and in Fig. 4a and c, where distinct permittivity values were obtained (*i.e.*, PT 3 > PT 2 > PT 1 and BT 3 > BT 2 > BT 1), while the *d*_33_ values were kept the same (*i.e.*, PT 3 ≈ PT 2 ≈ PT 1 ≈ 490 pC N^−1^ and BT 3 ≈ BT 2 ≈ BT 1 ≈ 330 pC N^−1^), respectively, considering the allowed 10% system error introduced by the Berlincourt meter used to measure the *d*_33_ values.^55,56^ The dielectric losses were below 4% and the values were comparable among the ceramics at specific frequencies (Fig. 3b and 4b). The low dielectric losses also ensured proper poling of the ceramics. Therefore, the measured ceramic dielectric and piezoelectric properties could be confidently treated as reliable estimates for the properties of the fillers during the electrical characterization of the corresponding composites.

In the composites, equalization of both the permittivity and dielectric loss values was seen at all frequencies for both the Pb-based and Pb-free samples (Fig. 3d, e and 4d, e), respectively, given that their deviations are considered. Consequently, the percentage of the composite relative permittivity (*ε*_c_) out of the ceramic relative permittivity (*ε*_p_), *i.e.*, (*ε*_c_/*ε*_p_)% in Fig. 3g and 4g, decreased almost linearly from PT 1 to PT 3 as well as from BT 1 to BT 3, indicating a lower retention of the permittivity in the composites as the ceramic values increased, respectively.

Clearly, this change from the ceramics to the composites is attributed to the concentration of the electric field in the binder during the dielectric measurement using the LCR method, wherein a larger number of dipoles were formed on the binder proportional to the *ε*_p_ value, with the relative permittivity of binder being constant among all the samples (≈5 at 1 kHz).^5^ Since the charge mobility was restricted in the binder, a larger portion of the measured *ε*_c_ was actually contributed from the binder and the interfacial polarizations generated, despite the much larger filler permittivity. Similarly, a larger contribution of the composite dielectric loss ((tan *δ*)_c_) stemmed from the binder (≈0.013 at 1 kHz^5^), thus rendering the contribution of dielectric loss from the filler to be negligible. Furthermore, a drastic difference between the *ε*_c_ values obtained in the relatively low frequency range (1–100 kHz) for the same sample was not noticeable, suggesting that this phenomenon was a true and universal effect.

However, it must be noted that the average *ε*_c_ and (tan *δ*)_c_ values decreased with an increase in frequency amongst the composite samples. The trend for *ε*_c_ was the same as that for the ceramics but the ceramic dielectric loss, (tan *δ*)_p_, showed the opposite trend. This suggests that there might be a mild opposing effect to the phenomenon of dominance of binder on electric field caused by the contribution of a greater amount of space charge polarization generated at the interfaces in the composite at lower frequencies.

The superposition of both the dominance of binder on electric field and the space charge polarization at filler-binder interface produced the trends seen in the ceramic to composite dielectric loss ratio, ((tan *δ*)_p_/(tan *δ*)_c_)%, as shown in Fig. 3h and 4h, respectively. At higher frequencies, larger ((tan *δ*)_p_/(tan *δ*)_c_)% values were observed, while within each composite type, the trends followed that of (tan *δ*)_p_ (Fig. 3b and 4b), respectively.

Despite these subtle observations regarding (tan *δ*)_c_, which could be associated with different phenomena, the (tan *δ*)_c_ values were generally very low (1.5–4%) and comparable between samples of the same type and at different frequencies. The low losses ensured proper poling in the composites and enabled a fair comparison of the piezoelectric properties among the composites as well as with their ceramic counterparts.

In Fig. 3f and 4f, a dramatic reduction in *d*_33_ for all the composite samples (*d*_33c_) compared to that of the corresponding ceramic samples (*d*_33p_) can be seen. This is a clear indication of the biasing effect seen on the electric field distribution in the composite during poling, where the magnitude of the electric field was proportionally more concentrated on the low-permittivity and non-piezoelectric (PTMA)CdCl_3_ binder^4^ as the filler permittivity increased.^19^

Importantly, in the case of the composite samples possessing a very large filler relative permittivity (*e.g.*, >1900) such as PT 2-C and PT 3-C, *d*_33c_ and (*d*_33c_/*d*_33p_)% attained constant values (Fig. 3f and i), respectively. This can be interpreted as an extreme case of the electric field biasing effect, where the filler and binder permittivities were so disparate that the electric field distributed on the fillers could no longer be reduced significantly, even though the filler permittivity kept increasing. This phenomenon can be further proven by the trend of *d*_33c_ and (*d*_33c_/*d*_33p_)% for the Pb-free composite samples (Fig. 4f and i), respectively. While the PT 1-C *d*_33_ value was unambiguously larger than that of PT 2-C and PT 3-C, the *d*_33_ values of BT 1-C, BT 2-C and BT 3-C were barely different, with the average value of BT 1-C being only slightly higher. The deviations were seen to overlap amongst the samples, suggesting that saturation of this dominating effect was attained for all the Pb-free samples due to their larger filler permittivity than that of the Pb-based samples. However, it should be noted that the lower *d*_33_ of the Pb-free ceramics than that of the Pb-based ones also contributes to the relatively higher tendency for plateauing in the *d*_33_ of their composites.

Given that the Berlincourt meter was considered less accurate than the impedance analyzer for judging the piezoelectricity, more proof is provided in Fig. S15 and S16 in the SI to consolidate the observation. The variation in *Θ* with respect to the measurement frequency is shown, and the maximum phase angle difference between the baseline and the first harmonic (Δ*Θ*_max_) is compared within the poled Pb-based samples and within the poled Pb-free samples. The value of Δ*Θ*_max_ indicates the poling efficiency, and hence gives an alternative quantification to the strength of the piezoelectric response in a certain sample.^53^

According to Fig. S15d and S16d, the evolution of Δ*Θ*_max_ followed the same trend as that of the *d*_33c_ and (*d*_33c_/*d*_33p_)%, *i.e.*, PT 1-C (Δ*Θ*_max_ ≈ 7.7°) > PT 2-C (Δ*Θ*_max_ ≈ 4.9°) ≈ PT 3-C (Δ*Θ*_max_ ≈ 4.1°) and BT 1-C (Δ*Θ*_max_ ≈ 1.2°) ≈ BT 2-C (Δ*Θ*_max_ ≈ 1.1°) ≈ BT 3-C (Δ*Θ*_max_ ≈ 0.8°). This observation helped to confirm that when the filler permittivity became sufficiently large, the electric field flux seemed to equilibrate in the fillers of these composites rather than continuously decreasing, indicating a parabolic relationship between the filler permittivity and *d*_33c_. Saturation of the electric field in the low-permittivity binder could be the cause of this.^19^ The modelling results in Section 3.4 well explain this behavior.

To validate these conclusions further, the effective electric field on the fillers based on an externally applied field was investigated in terms of the magnitude as well as transient characteristics, since an alternating current (AC) electric field was applied during the dielectric measurement as opposed to the steady direct current (DC) field applied during poling. An analytical equation proposed in the literature^54^ describing the transient response of the ferroelectric fillers in a 0–3 connected composite, such as in our case, was solved for this purpose in Section 8S.1 of the SI, using the data for the Pb-based samples because they display more apparent trends of *ε*_c_ and *d*_33c_.

The permittivity and conductivity (*σ*) (Table S16) of the phases in the composites determined the corresponding relaxation time (*τ*) of the interfacial polarization (Table S17), which further influenced the magnitude of the electric field on the fillers. Furthermore, specific to upside-down composites with large filler volume fractions, the ‘clustering effect’ of fillers focused the electric field on the numerous amounts of neck regions present between closely contacting fillers.^57^ As a result, the experimental relaxation times were possibly even higher than the calculated ones in Table S17 for a larger filler permittivity. Therefore, the periods of external field application during LCR measurement as well as during poling proved to be much shorter compared to the relaxation times in the composites. In this case, the electric fields on the filler varied inversely with the disparateness in the permittivity between the filler and binder, concurrent to the experimental observations in the Pb-based samples.

The saturation phenomena of the composite permittivity and *d*_33_ observed in Fig. 3d, f and 4d, f were further investigated in Section 8S.2 in the SI, respectively. The intrinsic physical mechanism of this behavior is described as a build-up of polarization in the low-permittivity binder, leading to a smaller penetration depth of the electric field into the fillers^19,58^ due to the differing work functions between the binder and filler phases. This mechanism can also be justified by considering each filler-binder interface as a small capacitor (Fig. S17) that can be extended to the entire composite based on an equivalent circuit, where the filler and binder are electrically equivalent to a closed circuit of capacitors connected in series, as shown in Fig. S18. Thus, there is an inverse distribution of voltage in each phase based on their capacitances, which are nominally the permittivity of the filler and binder, respectively.^26^ Complete negation of this field occurs over very large filler permittivity values, producing saturation in *ε*_c_ and *d*_33c_. This was also confirmed through finite elemental modelling of composites with similar connectivity in the literature.^57^ These analyses can be extended to the Pb-free samples analogously.

### 3.4. Modelling of upside-down composites

#### 3.4.1 Fitting electrical properties to available models

To further understand the upside-down composites in a more fundamental way, a series of commonly used/researched models was firstly screened by fitting the experimental dielectric data of the composites fabricated in this work to models including the Maxwell-Garnett model,^59^ the Yamada model,^60^ the Lichtenecker model and its modified version,^43,61^ the Poon model,^62^ and the Jayasundere model.^63^ Moreover, the composite permittivity predicted by each model was then fed to the Yamada model^60^ to predict *d*_33_. The best possible fit for each model against the experimental data was achieved *via* the least squares method by appropriately changing the fitting constants wherever applicable.

Fig. S19 and S20 in the SI compare the fitting results and Table S18 lists the obtained errors of the best fitting results against the experimental data. Comparing the errors for both the permittivity and *d*_33_, the Lichtenecker model was selected for describing the permittivity, whilst the Yamada model fed by the Lichtenecker dielectric predictions was used for describing the *d*_33_, as this solution showed the least simultaneous errors among the models during the fitting. Moreover, the selected models were applicable for biphasic composites consisting of isotropic fillers distributed in a homogenous binder with relatively high filler volume fractions, which is the closest description in existing models for the microstructure of the upside-down composites studied in this work.

As has been defined in eqn (4) and (5), *k* in the Lichtenecker model and *n* and *α* in the Yamada model were used as the fitting parameters given the input parameters such as *ε*, *φ*, and *d*_33f_. The use of *k*, *n*, and *α* was beneficial because they not only gave the most accurate fits but also defined the microgeometry, effects of size and orientation of the fillers on the composite properties, and the extent of polarization in the composite, respectively. These definitions facilitate the fundamental understanding of the composites in combination with the experimental data.

It should be noted that these constants have specific boundary conditions based on the assumptions used when deriving eqn (4) and (5). The value of *k* is constrained by the Wiener boundary conditions, *i.e.*, −1 < *k* < 1. *k* = 1 and *k* = −1 indicate filler particles being aligned in parallel with (parallel connection) and perpendicular to (series connection) the applied electric field during the permittivity measurement, respectively. *k* = 0 indicates evenly distributed particles without parallel or perpendicular orientations. *n* = 3 indicates spherical filler particles with no orientational effects, whilst *n* > 3 indicates the extent of filler elongation along the 3-direction after poling. *α* = 1 represents the theoretical maximum effectiveness of domain reorientation after poling, which is typically achieved by extensive optimization of the poling conditions including poling time, temperature, and electric field.^64,65^

**Fig. 5:**
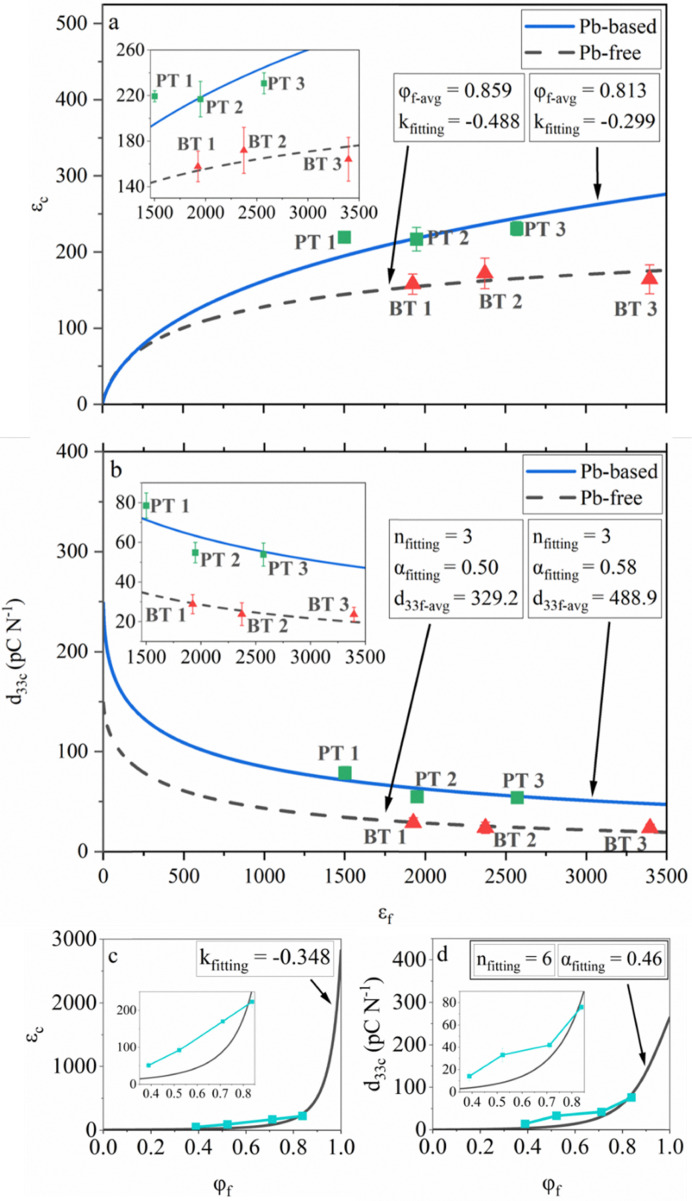
Dependence of (a) and (c) unpoled *ε*_c_ and (b) and (d) *d*_33c_ after poling on (a) and (b) filler relative permittivity (*ε*_f_) and (c) and (d) filler volume fraction (φ_f_) obtained from the fitting results of the (a) and (c) Lichtenecker model and (b) and (d) Yamada model, fed by the dielectric predictions from the Lichtenecker model, and for the experimental data (a) and (b) obtained in this work and (c) and (d) extracted for another PZ29-(PTMA)CdBr_1.5_Cl_1.5_ upside-down composite from a reference dataset reported in a previous work, respectively.^5^ The unit of *d*_33f-avg_ is pC N^−1^.

Fig. 5a and b show the optimized fitting results for the unpoled *ε*_c_ and *d*_33c_ after poling, respectively. The values of the fitting constants (*k*_fitting_, *n*_fitting_ and *α*_fitting_) extrapolated using the least squares method are listed alongside. The input parameters, *φ*_f-avg_ and *d*_33f-avg_, were taken from the experimental data by averaging the volume fractions of the filler (Table 1) and *d*_33_ of the ceramic, respectively, of the corresponding Pb-based or Pb-free samples. The relative permittivity of the binder (*ε*_b_) was taken as 5.^5^ Other relevant dielectric properties were taken at 1 kHz from the experimental data.

Satisfactory fits between the models and the experimental observations were achieved for both sets of composites when allowing deviations of the experimental data. The dominance of binder on electric field demonstrated as saturation/equalization of the composite permittivity with an increase in filler permittivity, as shown in Fig. 3 and 4, became evident in the modelling when viewing the parabolicity and asymptotic nature of the predicted curves towards plateaus for both sets of composites, respectively. This nature was more pronounced in the Pb-free composites owing to their generally higher filler permittivity. The asymptotic minimum value indicates the maximum extent of the dominance of binder on poling electric field, beyond which a constant value of *d*_33_ was obtained in the composites.

The PT 1-C sample was found as a minor outlier in Fig. 5a, which was underestimated by the model. Given that the electric field was mostly concentrated in the vicinity of the filler, and given the high filler volume fraction as well as the lower dominance by the binder on electric field owing to the lower filler permittivity (≈1500 in PT 1-C compared to >1900 in the other samples), a percolation threshold of electric field permeation inside the composite could have been reached.^66,67^ Consequently, the small electric field (1 V, equivalent to <1 V mm^−1^) supplied by the LCR meter during the dielectric measurement was able to polarize a larger volume of the filler compared to the case in other composites, yielding a greater experimental value that was not accounted for by the Lichtenecker model.

Another probable reason could be the effect of the spontaneous strain generated in the fillers during the paraelectric–ferroelectric phase transition occurring across the Curie temperature (*T*_C_)^68,69^ in the cooling stage during the fabrication of the composites. For instance, the *T*_C_ of the PT 1, PT 2, and PT 3 fillers was approximately 235 °C,^70^ 200 °C,^71^ and 120 °C,^72^ respectively. Empirically, the pressure applied during the fabrication of the composite (250 MPa) was able to decrease the phase transition temperature by roughly 50 °C.^5^ Therefore, by simultaneously elevating the temperature to 150 °C alongside the pressure during fabrication, the PT 2 and PT 3 fillers being at or in the vicinity of their *T*_C_ transformed either fully or at least partially into their paraelectric cubic phases. Upon cooling, the ferroelectric phases were restored but a spontaneous strain pinned by the (PTMA)CdCl_3_ binder could have been generated in the filler. Although the (PTMA)CdCl_3_ binder is known to help ease interfacial strain due to its ferroelastic nature,^5^ the extra strain in PT 2-C and PT 3-C could still relatively suppress their measured permittivity compared to PT 1-C, which did not go through a paraelectric–ferroelectric phase transition. Owing to this, the fitting based on the experimental results relatively underestimated the permittivity of PT 1-C.

A negative value of *k*_fitting_ was obtained for both the Pb-based (−0.299) and Pb-free (−0.488) composites. This is a consequence of the pressure-assisted densification method. A previous work on deformation experiments for polyphasic composite materials^73^ has suggested that during the initial compression of the composite and rearrangement of the filler particles inside, the connectivity of the harder filler components along the direction perpendicular to the flow of the binder compound can decrease. In the case of upside-down composites, a part of the binder could be squeezed out of the sides of the composite during the compression due to capillary forces. The binder could then flow out through the interfaces of the die-piston assembly. This phenomenon was confirmed in this work by observing the dimensions of each specimen, as a thickness gradient from the center to the sides was universally observed, contouring the flow of the binder which is due to the analogous pressure gradient from the center of the pressing piston to its sides when compressing the composite.^74^ Due to this lateral flow of the binder, the parallel connectivity of the fillers was likely to decrease, and consequently a more laterally linked filler microgeometry (transiting towards a series connection) was obtained, which was quantitatively described as a negative *k*_fitting_ value.

The *k*_fitting_ value for the Pb-based composites was larger than that of the Pb-free ones because the binder volume fractions were higher in the Pb-based composites (Table 1), which resulted in a larger number of filler-binder interfaces, and thus a smaller extent of series connection in the microgeometry of the Pb-based composites.

The value of *n*_fitting_ = 3, which implies spherically shaped filler particles, was considered realistic for this work. Even though the FESEM images (Fig. 2 and Fig. S3–S7) suggested irregularly shaped fillers at a local scale (particle size being only 0.1–1% of the dimensions of the specimen), since the ceramics were crushed under even pressures from the hydraulic press to produce the fillers, while no alignment of the fillers within themselves or in the composites was introduced, the filler particles could be globally treated as spheres. Thus, the irregularity of the shape and orientation of the fillers are randomized and effectively averaged out, as witnessed by the close fitting of the model to the experimental data.

The *α*_fitting_ values for the Pb-based (0.58) and Pb-free (0.50) composites were inferior to some other works (usually >0.8), which also used the Yamada model.^16,24,27,28^ This well reflected the fact that optimization of the poling conditions was not carried out in this work. The marginally higher *α*_fitting_ of the Pb-based composites than that of the Pb-free ones was owing to the presence of a larger amount of (PTMA)CdCl_3_ binder (Table 1). Therefore, more interfaces between the fillers were filled by a crystalline bridge of the binder, which enabled better electric field permeation between the fillers during poling.

Similar to *k*_fitting_, the *α*_fitting_ values could also be influenced by minor changes in the microgeometry induced by the changes in the binder volume fractions. It can be inferred that with high filler volume fractions where the interactions between the fillers were greatly enhanced due to the decrease in the inter-filler distance, even minor changes in the binder volume fractions, and hence the microgeometry can give substantial variations in the predictions.

To consolidate the perspective of the influence of solely an extended range in permittivity in the fillers, the relevant data from representative piezoceramics in the literature^75–82^ were extracted and modelled by assuming that those fillers were used in upside-down composites. Table S19 details the properties of these piezoceramics. Fig. S21 and S22 show the trends of the unpoled *ε*_c_ and the *d*_33c_ of the imagined composites. It is unambiguous that the same conclusions can be reached when *d*_33f_ was kept the same while the filler permittivity varied. As dictated by the models, piezoceramics with lower permittivity values tend to induce lower *ε*_c_ and higher *d*_33c_ and higher values tend to saturate these values. However, it should be noted that percolative effects similar to the case of PT 1-C might enable some of the upside-down composites made with low permittivity fillers to possess larger values of *ε*_c_ and *d*_33c_ compared to the prediction by the model, which can be investigated in future work.

It is evident that for improving the *d*_33_ in the composites, apart from reducing the mismatch in permittivity between the phases, the poling efficiency needs to be increased by optimizing the poling conditions (time, temperature and electric field) in future works.^9^ Moreover, alternative poling methodologies such as corona poling can be utilized for the composites, which provide several advantages over the conventional contact poling used in this work. These advantages include a lower chance of dielectric breakdown at the required poling fields, owing to the differing charge transfer mechanisms, where local short-circuits due to the aforementioned permittivity mismatch between the phases are prevented,^83^ and the elimination of the need for electrodes that are typically responsible for macroscopic short-circuits owing to the interfacial charges present.^84^ Furthermore, investigations towards using more conductive phases could be performed owing to the decreased relaxation times in the composites.

#### 3.4.2 Limitation of modelling

Fits for the experimental data collected in a previous work are shown in Fig. 5c and d, where varying filler volume fractions in PZ29 (filler)-(PTMA)CdBr_1.5_Cl_1.5_ (binder) upside-down composites were involved.^5^ This step was performed in an attempt to extend the models towards lower filler volume fractions, and thus bridge the knowledge gap existing in the transition region from upside-down to conventional composites.

However, the experimental data for *ε*_c_ and *d*_33c_ fitted to the models only at *φ*_f_ = 0.838, while other data with lower *φ*_f_ values were substantially underestimated in the models. The incorrect predictions should be attributed to the evaporation of the organic compounds from the binder at the fabrication temperature (≈150 °C), which was aggravated by the applied pressure.^5^ As a result, the actual *φ*_f_ became higher than the designed value, leading to higher actual *ε*_c_ and *d*_33c_ than the predictions. The larger the binder volume was in the composites, the more likely the experimental data were affected by evaporation due to the competing capillary force that keeps the binder inside the composite with the free energy for the binder to evaporate. In addition, the above-mentioned binder being squeezed out during fabrication also contributed to the failure of the model when predicting the properties of the upside-down composites with a lower *φ*_f_.

Here, this study faces a challenge. To further optimize the functional properties of upside-down composites, appropriate models should be found to correctly describe their microstructure–property correlations, and thus to guide the material design. However, the feature of the pressure-assistance in the ultra-low-temperature densification method inevitably creates deviations in the actual microstructure from the design, imposing difficulties, and thus necessity of finding broadly applicable models.

## 4. Conclusions

This work used a combination of models (Lichtenecker and Yamada) and experiments to validate the dominance of binder on the electric field and its saturation in oxide-halide perovskite upside-down composites made from a low-permittivity binder and high-volume, high-permittivity fillers. The experimental filler properties have been specially controlled, where two sets of fillers, Pb-based and Pb-free, which possess the same *d*_33_ but vastly different permittivity within each set, were used to fabricate the composites. Despite the distinctive filler permittivity, the composite permittivity within each set of samples has been found to be similar. Alternatively, the composite piezoelectric properties characterized by *d*_33_ and impedance analysis exhibit a counter trend with respect to the corresponding filler permittivity.

These results, strengthened by the models simultaneously, prove that the magnitude of electric field flowing in the composite during poling or LCR measurement is proportionally more concentrated on the low-permittivity and non-piezoelectric binder with an increase in the filler permittivity. The high filler volume fractions in the composites, which induce a ‘clustering effect’, cause larger relaxation times of the interfacial polarization, adding an additional impact to this effect. Interestingly, it is also found that for composites with sufficiently high filler permittivity, the biasing effect of electric field in the binder seems to saturate, and thus constant dielectric and piezoelectric properties are attained regardless of a further increase in the filler permittivity. Although this conclusion could have been predicted in models, it is the first time that this saturation effect has been experimentally visualized in upside-down composites. The physical mechanism behind this effect is attributed to the eventual negation of the electric field in the fillers with an increase the filler permittivity.

The understanding of the microgeometry, shape and orientation of the fillers, and poling efficiency with the help of both modelling and experiments in this work will guide future recycling works on piezoceramics for second-life applications. Nevertheless, the validity of the models is questioned when extended to lower filler volume fractions due to the evaporation of the binder in reality under the pressure-assisted, ultra-low-temperature densification method. This fact challenges the attempt of bridging the gap in the transition region from upside-down to conventional composites.

Future research should investigate this issue by developing predictive methodologies that are superior to the currently used procedure of fitting analytical models to experiments, which is valid only for certain boundary conditions. A more fundamental study of upside-down composites in terms of the relationships among the fitting parameters, fabrication conditions, input parameters, and resulting functionalities is necessary. In addition, possible developments may point towards using the upside-down compositing method for organic–inorganic hybrid energy storage materials and photopolymerization in 3D printing technology for piezoelectric ceramics.

Towards upscaling the recycling process in an industrial context, the foremost step would be to perform a lifecycle assessment of the upside-down compositing method starting from the extraction of ceramics from discarded devices to the end of their proposed second life, keeping in mind potential trade-offs between incurred energy footprint and efficacy of recycling. Subsequently, the methodology should be optimized for industrial operation. This would necessitate two separate branches of study. The first would be to optimize the dielectric and piezoelectric properties of the recycled materials to satisfy the requirements of the majority of piezoelectric applications. For instance, composites made from other filler and binder combinations and with better poling efficiencies can be investigated, so that they cover a broader range of applications for potential second lives. The processing of fillers such as their crushing methods could also be investigated as they are likely to play a role in determining the composite properties, as is predicted by the shape anisotropy parameter in the Yamada model. The second would be to upgrade the modelling procedure towards seamless prediction of composite functionality, even under complex instances, such as when recycling multiple fillers that cannot be separated, and/or when the fillers are mixed with additives or electrodes (very likely to happen for recycled industrial products). Ideally, the predictive method should be compatible with other potential recycling techniques without the need for another tedious cycle of experimental trial and error. A possible solution is to use high-throughput experimental testing methods in collaboration with AI-assisted modelling, which can handle and analyse large datasets to suggest the best possible approach for a particular application.

## Author contributions

Sivagnana Sundaram Anandakrishnan: data curation, formal analysis, investigation, visualization, and writing – original draft. Mikko Nelo: methodology, supervision, writing – review and editing. Mohadeseh Tabeshfar: investigation and methodology. Viktoria Kraft: investigation and methodology. Neamul Hayet Khansur: resources, writing – review and editing. Jani Peräntie: supervision, writing – review and editing. Yang Bai: conceptualization, funding acquisition, methodology, project administration, resources, supervision, validation, writing – review and editing.

## Conflicts of interest

The authors declare no conflict of interest.

## Supplementary Material

MA-006-D5MA00554J-s001

## Data Availability

The original and raw data supporting the findings of this study are openly available in Fairdata.fi Etsin at DOI: https://doi.org/10.23729/fd-81143940-a78f-3027-8507-8fc458026b59, reference number.^32^ For extra details that enhance the clarity of the results. See DOI: https://doi.org/10.1039/d5ma00554j
