# Supplementary material for: Influence of the permittivity between fillers and binders on the properties of upside-down composites for recycling purposes
Source: Mater Adv. 2025 Jul 28;6(19):6694–710. doi: 10.1039/d5ma00554j (PMC12334081; doi:10.1039/d5ma00554j)
Supplement: MA-006-D5MA00554J-s001 [file MA-006-D5MA00554J-s001.pdf]

Supplementary Information (SI) for Materials Advances.
This journal is © The Royal Society of Chemistry 2025

Supporting Information

Influence of permittivity between fillers and binders on properties of upside-down composites for recycling purpose

Sivagnana Sundaram Anandakrishnan[1,2], Mikko Nelo[1,2], Mohadeseh Tabeshfar[1,2], Viktoria Kraft[3], Neamul Hayet Khansur[3,4], Jani Peräntie[1], and Yang Bai[1]*

[1]Microelectronics Research Unit, Faculty of Information Technology and Electrical Engineering, University of Oulu, FI-90570 Oulu, Finland

[2]Infotech Oulu, FI-90570 Oulu, Finland

[3]Department of Materials Science and Engineering, Friedrich-Alexander-Universität Erlangen-Nürnberg (FAU) 91058 Erlangen, Germany

[4]Department of Materials Science and Engineering, Case Western Reserve University, Cleveland, OH 44106, USA

*Corresponding author: yang.bai@oulu.fi

1S. Details of fabricated ceramic and composite samples

The dimensions of all the prepared samples and the amount of fillers used for each composite sample are listed in Table S1. Photographs of representative specimens from each type of samples are shown in Figure S1.

Table S1. List of filler amount and dimensions of the samples fabricated in this work

Sample ID	Sample description	Amount of filler used in each composite sample (g)	Thickness (mm)	Diameter (mm)
PT 1-P	PZ29 ceramic	-	0.904 ± 0.002	8.18 ± 0.01
PT 2-P	APC-855 ceramic	-	0.827 ± 0.002	7.92 ± 0.005
PT 3-P	$0.67Pb(Mg_{1/3}Nb_{2/3})O_3$-$0.33PbTiO_3$ ceramic	-	2.333 ± 0.002	8.46 ± 0.01
BT 1-P	$(Ba_{0.80\pm0.02}Ca_{0.14\pm0.005})(Ti_{0.90\pm0.005}Zr_{0.10\pm0.005})O_{2.94\pm0.02}$ ceramic	-	0.955 ± 0.003	8.20 ± 0.01
BT 2-P	$(Ba_{0.95\pm0.01})(Ti_{0.94\pm0.005}Sn_{0.06\pm0.005})O_{2.95\pm0.01}$ ceramic	-	1.157 ± 0.002	8.50 ± 0.01
BT 3-P	$(Ba_{0.79\pm0.01}Ca_{0.14\pm0.005})(Ti_{0.88\pm0.005}Zr_{0.12\pm0.005})O_{2.94\pm0.02}$ ceramic	-	0.970 ± 0.003	8.23 ± 0.01
PT 1-C	Upside-down composite	0.6	1.324 ± 0.032	10.02 ± 0.01
PT 2-C	Upside-down composite	0.6	1.293 ± 0.031	10.01 ± 0.01
PT 3-C	Upside-down composite	0.6	1.288 ± 0.040	10.01 ± 0.01
BT 1-C	Upside-down composite	0.6	1.624 ± 0.026	9.99 ± 0.02
BT 2-C	Upside-down composite	0.6	1.528 ± 0.018	9.98 ± 0.02
BT 3-C	Upside-down composite	0.6	1.602 ± 0.020	9.99 ± 0.01

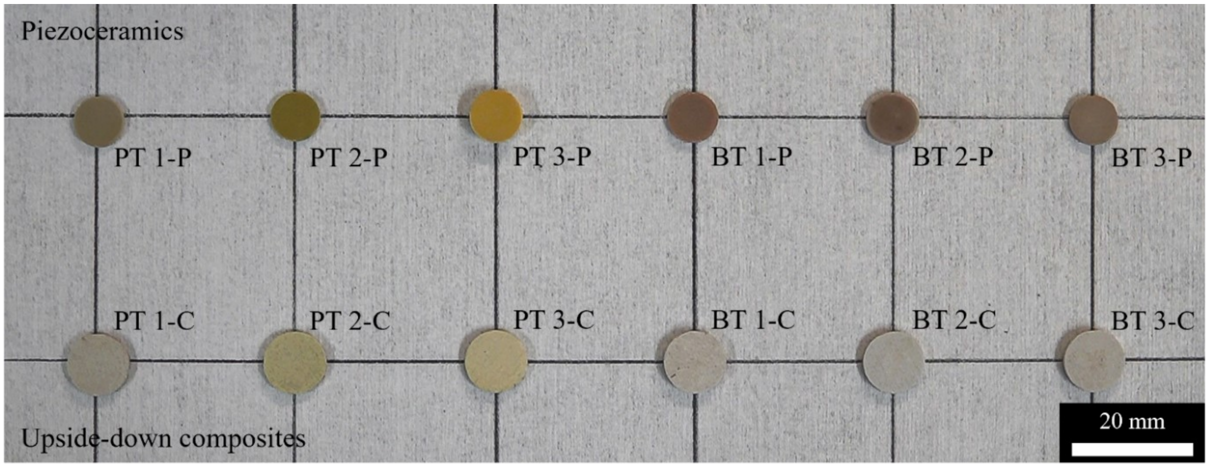

Figure S1. Photographs of example specimens from the ceramic and composite samples prepared in this work.

2S. XRD (X-ray diffraction) results of ceramic fillers

The Rietveld refinement results as well as the obtained fitting parameters are shown in Figure S2. Rietveld refinement was carried out in the SmartLab Studio-II software. Diffraction data of representative phases under Cu-Kα radiation were acquired from the ICDD (International Center for Diffraction Data) database using the PDF-5+ software and matched to the experimentally obtained diffraction profile of the ceramics in this work. The Rietveld refinement was carried out using the d-I pattern method, on the scale, lattice, profile, and background of the diffraction data, while the texture and structure parameters were fixed. The cycle count was restricted to 20. The functions used for refining the background, peak shift, and profile were the B-spline, shift-axial displacement, and split pseudo-Voigt, respectively. Lorentz, polarization, and slit/absorption/asymmetric corrections were applied, whilst restraints and occupancies were not set during the refinement. The fitting parameters were obtained based on the closeness of the fitted profile to that of the experimentally observed one.

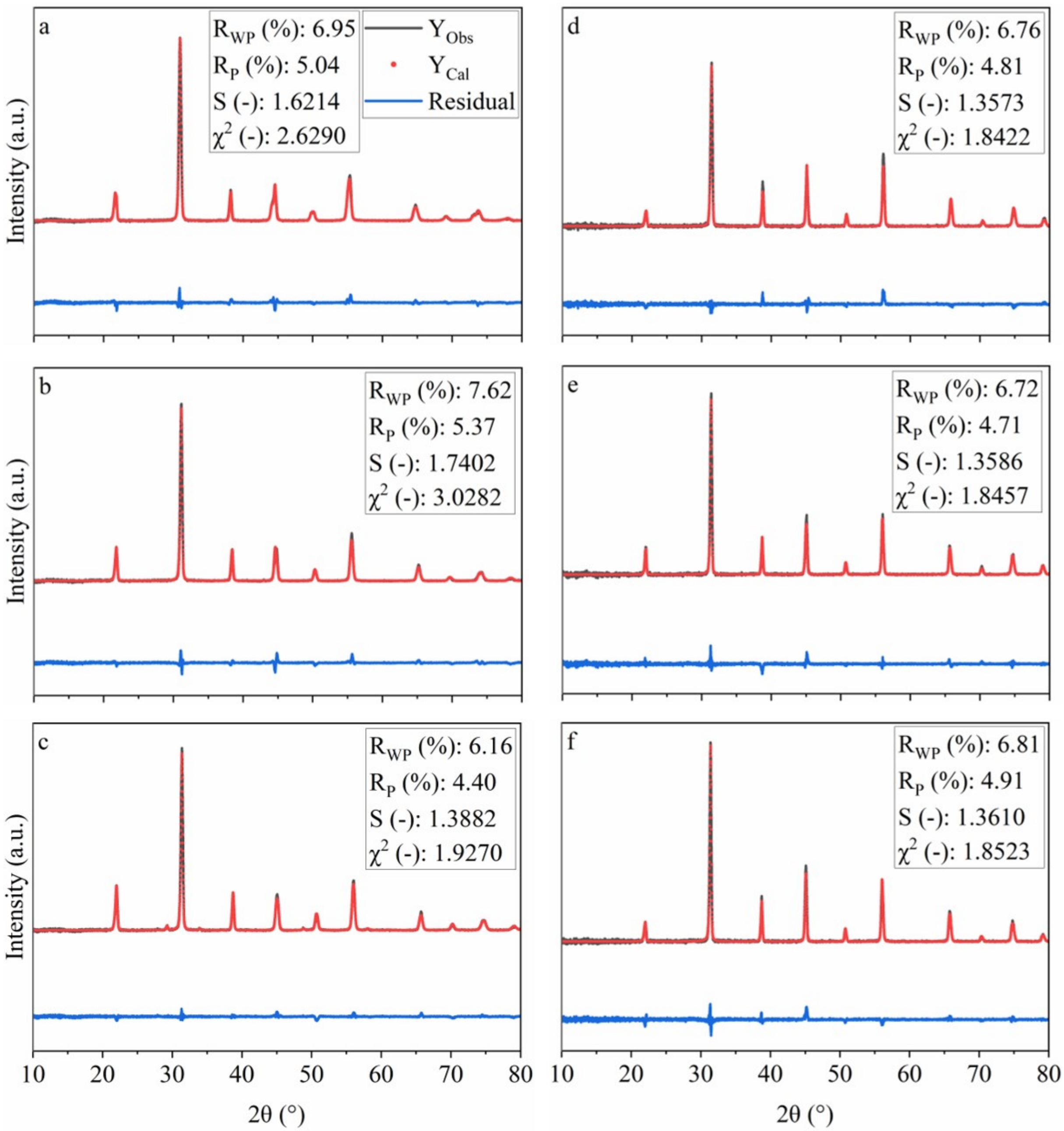

Figure S2: Observed XRD reflections (Y_{obs}), calculated curves (Y_{cal}), their respective residual errors after Rietveld refinement, and the associated refinement parameters for (a) PT 1, (b) PT 2, (c) PT 3, (d) BT 1, (e) BT 2, and (f) BT 3, respectively.

Table S2. List of XRD reflections for PT 1

2θ (°)	Intensity (normalized)	d-space (Å)	Diffraction plane
21.65	9.9	4.1008	P4mm (0 0 1)
21.87	17.3	4.0613	P4mm (1 0 0)
30.96	100.0	2.8856	P4mm (1 0 1)
31.12	52.0	2.8718	P4mm (1 1 0)
38.23	21.1	2.3523	P4mm (1 1 1)
44.13	10.2	2.0504	P4mm (0 0 2)
44.59	26.2	2.0306	P4mm (2 0 0)
49.78	4.6	1.8303	P4mm (1 0 2)
50.09	2.9	1.8198	P4mm (2 0 1)
50.19	2.6	1.8163	P4mm (2 1 0)
54.98	12.6	1.6687	P4mm (1 1 2)
55.27	25.2	1.6607	P4mm (2 1 1)
64.54	7.3	1.4428	P4mm (2 0 2)
64.89	4.5	1.4359	P4mm (2 2 0)
68.60	0.2	1.3669	P4mm (0 0 3)
69.02	1.7	1.3596	P4mm (2 1 2)
69.28	0.4	1.3552	P4mm (2 2 1)
72.97	2.9	1.2955	P4mm (1 0 3)
73.63	3.0	1.2855	P4mm (3 0 1)
73.71	3.1	1.2843	P4mm (3 1 0)
77.23	0.4	1.2342	P4mm (1 1 3)
77.88	1.1	1.2256	P4mm (3 1 1)

Table S3. Phase information of the assigned phase for the PT 1 filler based on the XRD reflections

**Parameter**	**Primary phase (♦)**
Chemical formula	$O_3\ Pb_{0.91}\ Sr_{0.06}\ Ti_{0.5}\ Zr_{0.5}$
Composition	$O_3\ Pb_{0.91}\ Sr_{0.06}\ Ti_{0.5}\ Zr_{0.5}$
Z-value	1
Concentration (%)	100
RIR value	10.631
DB card no.	01-070-6380
Reference	01-070-6380
Crystal system	Tetragonal
Space group	99 : P4mm
a (ang.)	4.0613
b (ang.)	4.0613
c (ang.)	4.1008
α (ang.)	90
β (ang.)	90
γ (ang.)	90
Calculated density (g cm^{-3})	7.64383
Mass absorption coefficient ($cm^2\ g^{-1}$)	168.246
Phase reg. method	ICDD (PDF 4+-2023)
Molar ratio (%)	100

Table S4. List of XRD reflections for PT 2

2θ (°)	Intensity (normalized)	d-space (Å)	Diffraction plane
21.90	18.1	4.0546	P4mm (1 0 0)
22.03	9.0	4.0320	P4mm (0 0 1)
31.17	50.3	2.8670	P4mm (1 1 0)
31.26	100.0	2.8590	P4mm (1 0 1)
38.50	26.4	2.3366	P4mm (1 1 1)
44.66	26.6	2.0273	P4mm (2 0 0)
44.93	12.6	2.0160	P4mm (0 0 2)
50.28	3.9	1.8133	P4mm (2 1 0)
50.34	4.0	1.8112	P4mm (2 0 1)
50.52	4.2	1.8052	P4mm (1 0 2)
55.52	29.4	1.6537	P4mm (2 1 1)
55.69	14.1	1.6491	P4mm (1 1 2)
65.01	5.1	1.4335	P4mm (2 2 0)
65.21	10.1	1.4295	P4mm (2 0 2)
69.49	0.2	1.3515	P4mm (3 0 0)
69.54	0.8	1.3507	P4mm (2 2 1)
69.69	2.1	1.3482	P4mm (2 1 2)
73.85	3.9	1.2822	P4mm (3 1 0)
73.90	3.9	1.2815	P4mm (3 0 1)
74.29	3.7	1.2757	P4mm (1 0 3)
78.16	2.4	1.2219	P4mm (3 1 1)
78.54	1.1	1.2169	P4mm (1 1 3)

Table S5. Phase information of the assigned phase for the PT 2 filler based on the XRD reflections

**Parameter**	**Primary phase (¤)**
Chemical formula	$Mg_{0.23}$ $Nb_{0.47}$ O_3 Pb $Ti_{0.14}$ $Zr_{0.16}$
Composition	$Mg_{0.23}$ $Nb_{0.47}$ O_3 Pb $Ti_{0.14}$ $Zr_{0.16}$
Z-value	1
Concentration (%)	100
RIR value	12.472
DB card no.	04-023-9159
Reference	04-023-9159
Crystal system	Tetragonal
Space group	99 : P4mm
a (ang.)	4.0546
b (ang.)	4.0546
c (ang.)	4.0320
α (ang.)	90
β (ang.)	90
γ (ang.)	90
Calculated density (g cm^{-3})	8.16056
Mass absorption coefficient (cm^2 g^{-1})	168.087
Phase reg. method	ICDD (PDF 4+-2023)
Molar ratio (%)	100

Table S6. List of XRD reflections for PT 3

2θ (°)	Intensity (normalized)	d-space (Å)	Diffraction plane
22.03	10.4	4.0307	P4mm (0 0 1)
22.05	25.2	4.0285	P1m1 (0 0 1)
22.13	22.5	4.0145	P1m1 (1 0 0)
22.14	20.1	4.0110	P4mm (1 0 0)
22.21	21.2	3.9992	P1m1 (0 1 0)
28.09	0.1	3.1736	Fd-3m (3 1 1)
29.37	6.7	3.0385	Fd-3m (2 2 2)
31.41	52.6	2.8458	P1m1 (1 0 -1)
31.44	51.6	2.8431	P4mm (1 0 1)
31.46	49.7	2.8414	P1m1 (1 0 1)
31.50	100.0	2.8382	P1m1 (0 1 1)
31.52	25.6	2.8362	P4mm (1 1 0)
31.55	99.1	2.8332	P1m1 (1 1 0)
34.04	2.2	2.6314	Fd-3m (4 0 0)
37.20	0.3	2.4148	Fd-3m (3 3 1)
38.79	14.0	2.3195	P4mm (1 1 1)
38.81	30.3	2.3186	P1m1 (1 1 -1)
38.85	34.8	2.3163	P1m1 (1 1 1)
44.70	0.1	2.0257	Fd-3m (5 1 1)
44.94	5.9	2.0154	P4mm (0 0 2)
44.97	28.4	2.0143	P1m1 (0 0 2)
45.13	29.8	2.0072	P1m1 (2 0 0)
45.18	11.9	2.0055	P4mm (2 0 0)
45.32	31.2	1.9996	P1m1 (0 2 0)
48.91	1.8	1.8607	Fd-3m (4 4 0)

50.63	4.8	1.8014	P1m1 (1 0 -2)
50.65	2.6	1.8008	P4mm (1 0 2)
50.70	4.2	1.7992	P1m1 (1 0 2)
50.71	8.8	1.7990	P1m1 (0 1 2)
50.74	3.0	1.7977	P1m1 (2 0 -1)
50.81	2.5	1.7955	P4mm (2 0 1)
50.81	3.5	1.7955	P1m1 (2 0 1)
50.86	6.9	1.7939	P1m1 (2 1 0)
50.86	2.4	1.7938	P4mm (2 1 0)
50.94	6.9	1.7911	P1m1 (0 2 1)
50.98	6.3	1.7898	P1m1 (1 2 0)
55.92	7.3	1.6428	P4mm (1 1 2)
55.94	17.2	1.6425	P1m1 (1 1 -2)
56.00	16.9	1.6408	P1m1 (1 1 2)
56.04	19.3	1.6396	P1m1 (2 1 -1)
56.07	14.7	1.6388	P4mm (2 1 1)
56.10	17.5	1.6380	P1m1 (2 1 1)
56.18	17.8	1.6361	P1m1 (1 2 -1)
56.21	17.0	1.6353	P1m1 (1 2 1)
58.08	1.5	1.5868	Fd-3m (6 2 2)
60.93	0.4	1.5193	Fd-3m (4 4 4)
65.55	4.8	1.4229	P1m1 (2 0 -2)
65.62	6.5	1.4216	P4mm (2 0 2)
65.67	5.3	1.4207	P1m1 (2 0 2)
65.75	10.5	1.4191	P1m1 (0 2 2)
65.80	3.3	1.4181	P4mm (2 2 0)
65.88	11.0	1.4166	P1m1 (2 2 0)

69.96	0.3	1.3436	P4mm (0 0 3)
70.01	1.4	1.3428	P1m1 (0 0 3)
70.14	2.1	1.3406	P1m1 (2 1 -2)
70.18	2.1	1.3399	P4mm (2 1 2)
70.25	2.0	1.3388	P1m1 (2 1 2)
70.27	2.0	1.3384	P1m1 (1 2 -2)
70.29	0.9	1.3382	P1m1 (3 0 0)
70.32	1.0	1.3377	P4mm (2 2 1)
70.33	1.9	1.3375	P1m1 (1 2 2)
70.36	0.5	1.3370	P4mm (3 0 0)
70.37	1.4	1.3368	P1m1 (2 2 -1)
70.42	1.6	1.3359	P1m1 (2 2 1)
70.60	0.7	1.3331	P1m1 (0 3 0)
71.67	0.1	1.3157	Fd-3m (8 0 0)
74.40	2.0	1.2741	P1m1 (1 0 -3)
74.40	3.4	1.2740	P4mm (1 0 3)
74.47	3.7	1.2730	P1m1 (0 1 3)
74.48	1.8	1.2729	P1m1 (1 0 3)
74.64	2.3	1.2705	P1m1 (3 0 -1)
74.72	2.1	1.2694	P1m1 (3 0 1)
74.75	3.4	1.2690	P4mm (3 0 1)
74.75	3.8	1.2690	P1m1 (3 1 0)
74.79	3.4	1.2684	P4mm (3 1 0)
74.99	4.0	1.2656	P1m1 (0 3 1)
75.02	4.0	1.2651	P1m1 (1 3 0)
78.75	1.7	1.2142	P4mm (1 1 3)
78.77	1.3	1.2139	P1m1 (1 1 -3)

78.85	1.7	1.2129	P1m1 (1 1 3)
79.01	1.1	1.2109	P1m1 (3 1 -1)
79.09	3.1	1.2099	P4mm (3 1 1)
79.09	1.5	1.2099	P1m1 (3 1 1)
79.28	0.3	1.2074	Fd-3m (6 6 2)
79.30	1.3	1.2072	P1m1 (1 3 -1)

Table S7. Phase information of the assigned phase for the PT 3 filler based on the XRD reflections

**Parameter**	**Primary MPB phase (★)**	**Primary MPB phase (#)**	**Secondary phase (*)**
Chemical formula	$Mg_{0.227}$ $Nb_{0.453}$ O_3 Pb $Ti_{0.32}$	$Mg_{0.22}$ $Nb_{0.43}$ O_3 Pb $Ti_{0.35}$	Nb_2 $O_{6.26}$ $Pb_{1.45}$
Composition	$Mg_{0.227}$ $Nb_{0.453}$ O_3 Pb $Ti_{0.32}$	$Mg_{0.22}$ $Nb_{0.43}$ O_3 Pb $Ti_{0.35}$	Nb_2 $O_{6.26}$ $Pb_{1.45}$
Z-value	1	1	8
Concentration (%)	82.20	15.40	2.41
RIR value	5.966	12.023	12.745
DB card no.	04-024-5195	04-026-8676	01-084-1731
Reference	04-024-5195	04-026-8676	01-084-1731
Crystal system	Monoclinic	Tetragonal	Cubic
Space group	6 : P1m1	99 : P4mm	227 : Fd-3m, choice-2
a (ang.)	4.0145	4.0110	10.5260
b (ang.)	3.9992	4.0110	10.5260
c (ang.)	4.0285	4.0307	10.5260
α (ang.)	90.000	90	90
β (ang.)	90.087	90	90
γ (ang.)	90.000	90	90
Calculated density (g cm^{-3})	8.16774	8.12403	6.68448
Mass absorption coefficient (cm^2 g^{-1})	170.829	171.227	156.8590
Phase reg. method	ICDD (PDF-4+ 2023)	ICDD (PDF-4+ 2023)	ICDD (PDF-4+ 2023)
Molar ratio (%)	83.10	15.60	1.32

Table S8. List of XRD reflections for BT 1

2θ (°)	Intensity (normalized)	d-space (Å)	Diffraction plane
22.18	18.6	4.0041	R3m:H (1 0 1)
31.57	98.8	2.8315	R3m:H (0 1 2)
31.58	100.0	2.8311	R3m:H (1 1 0)
38.92	10.6	2.3121	R3m:H (0 0 3)
38.93	30.4	2.3116	R3m:H (0 2 1)
45.26	75.1	2.0020	R3m:H (2 0 2)
50.95	7.3	1.7908	R3m:H (1 1 3)
50.96	5.7	1.7906	R3m:H (2 1 1)
56.22	16.3	1.6349	R3m:H (1 0 4)
56.23	36.1	1.6346	R3m:H (1 2 2)
56.23	18.4	1.6345	R3m:H (3 0 0)
65.92	14.8	1.4158	R3m:H (0 2 4)
65.94	15.2	1.4156	R3m:H (2 2 0)
70.49	0.8	1.3349	R3m:H (0 1 5)
70.50	0.4	1.3347	R3m:H (0 3 3)
70.50	0.6	1.3346	R3m:H (1 3 1)
74.94	8.8	1.2663	R3m:H (2 1 4)
74.95	9.2	1.2661	R3m:H (3 1 2)
79.28	1.0	1.2074	R3m:H (2 0 5)
79.29	2.3	1.2073	R3m:H (2 2 3)
79.30	0.5	1.2072	R3m:H (4 0 1)

Table S9. Phase information of the assigned phase for the BT 1 filler based on the XRD reflections

**Parameter**	**Primary phase (▼)**
Chemical formula	$Ba_{0.85}$ $Ca_{0.15}$ O_3 $Ti_{0.9}$ $Zr_{0.1}$
Composition	$Ba_{0.85}$ $Ca_{0.15}$ O_3 $Ti_{0.9}$ $Zr_{0.1}$
Z-value	3
Concentration (%)	100
RIR value	5.267
DB card no.	04-025-4917
Reference	04-025-4917
Crystal system	Trigonal (H)
Space group	160 : R3m:H
a (ang.)	5.6622
b (ang.)	5.6622
c (ang.)	6.9360
α (ang.)	90
β (ang.)	90
γ (ang.)	120
Calculated density (g cm^{-3})	5.76672
Mass absorption coefficient (cm^2 g^{-1})	219.5320
Phase reg. method	ICDD (PDF 4+-2023)
Molar ratio (%)	100

Table S10. List of XRD reflections for BT 2

2θ (°)	Intensity (normalized)	d-space (Å)	Diffraction plane
22.08	7.6	4.0223	P4mm (0 0 1)
22.14	14.6	4.0121	P4mm (1 0 0)
31.47	0.1	2.8408	Amm2 (1 1 1)
31.47	100.0	2.8406	P4mm (1 0 1)
31.51	49.8	2.8370	P4mm (1 1 0)
38.81	31.8	2.3183	P4mm (1 1 1)
45.04	16.0	2.0111	P4mm (0 0 2)
45.16	32.6	2.0060	P4mm (2 0 0)
50.74	4.0	1.7979	P4mm (1 0 2)
50.82	3.7	1.7952	P4mm (2 0 1)
50.85	3.6	1.7943	P4mm (2 1 0)
56.00	16.9	1.6407	P4mm (1 1 2)
56.08	34.2	1.6386	P4mm (2 1 1)
65.69	16.2	1.4203	P4mm (2 0 2)
65.78	8.2	1.4185	P4mm (2 2 0)
70.13	0.4	1.3408	P4mm (0 0 3)
70.25	2.9	1.3389	P4mm (2 1 2)
70.31	1.4	1.3377	P4mm (2 2 1)
70.34	0.7	1.3374	P4mm (3 0 0)
74.57	6.1	1.2716	P4mm (1 0 3)
74.74	6.2	1.2691	P4mm (3 0 1)
74.77	6.2	1.2687	P4mm (3 1 0)
78.91	2.7	1.2122	P4mm (1 1 3)
79.08	4.9	1.2100	P4mm (3 1 1)

Table S11. Phase information of the assigned phase for the BT 2 filler based on the XRD reflections

**Parameter**	**Primary MPB phase 1 (●)**	**Primary MPB phase 2 (■)**
Chemical formula	Ba O_3 $Sn_{0.06}$ $Ti_{0.94}$	Ba O_3 $Sn_{0.07}$ $Ti_{0.93}$
Composition	Ba O_3 $Sn_{0.06}$ $Ti_{0.94}$	Ba O_3 $Sn_{0.07}$ $Ti_{0.93}$
Z-value	2	1
Concentration (%)	0.6	99.4
RIR value	8.222	7.838
DB card no.	01-085-9628	04-007-5135
Reference	01-085-9628	04-007-5135
Crystal system	Orthorhombic	Tetragonal
Space group	38 : Amm2	99 : P4mm
a (ang.)	4.3070	4.0121
b (ang.)	6.0160	4.0121
c (ang.)	4.8580	4.0223
α (ang.)	90	90
β (ang.)	90	90
γ (ang.)	90	90
Calculated density (g cm^{-3})	6.27122	6.10797
Mass absorption coefficient (cm^2 g^{-1})	233.795	233.871
Phase reg. method	ICDD (PDF 4+-2023)	ICDD (PDF 4+-2023)
Molar ratio (%)	0.6	99.4

Table S12. List of XRD reflections for BT 3

2θ (°)	Intensity (normalized)	d-space (Å)	Diffraction plane
22.14	18.6	4.0114	R3m:H (1 0 1)
31.52	98.7	2.8361	R3m:H (0 1 2)
31.51	100.0	2.8369	R3m:H (1 1 0)
38.86	10.5	2.3153	R3m:H (0 0 3)
38.85	30.0	2.3162	R3m:H (0 2 1)
45.17	71.9	2.0057	R3m:H (2 0 2)
50.86	6.9	1.7938	R3m:H (1 1 3)
50.85	5.4	1.7942	R3m:H (2 1 1)
56.13	15.1	1.6373	R3m:H (1 0 4)
56.11	33.5	1.6377	R3m:H (1 2 2)
56.11	17.1	1.6379	R3m:H (3 0 0)
65.80	13.8	1.4181	R3m:H (0 2 4)
65.78	14.2	1.4185	R3m:H (2 2 0)
70.37	0.8	1.3368	R3m:H (0 1 5)
70.35	0.4	1.3372	R3m:H (0 3 3)
70.34	0.6	1.3373	R3m:H (1 3 1)
74.79	8.5	1.2684	R3m:H (2 1 4)
74.77	8.9	1.2686	R3m:H (3 1 2)
79.14	1.0	1.2093	R3m:H (2 0 5)
79.12	2.3	1.2095	R3m:H (2 2 3)
79.11	0.5	1.2097	R3m:H (4 0 1)

Table S13. Phase information of the assigned phase for the BT 3 filler based on the XRD reflections

**Parameter**	**Primary phase (▲)**
Chemical formula	$Ba_{0.85}$ $Ca_{0.15}$ O_3 $Ti_{0.9}$ $Zr_{0.1}$
Composition	$Ba_{0.85}$ $Ca_{0.15}$ O_3 $Ti_{0.9}$ $Zr_{0.1}$
Z-value	3
Concentration (%)	100
RIR value	5.268
DB card no.	04-025-4917
Reference	04-025-4917
Crystal system	Trigonal (H)
Space group	160 : R3m:H
a (ang.)	5.6738
b (ang.)	5.6738
c (ang.)	6.9460
α (ang.)	90
β (ang.)	90
γ (ang.)	120
Calculated density (g cm^{-3})	5.73505
Mass absorption coefficient (cm^2 g^{-1})	219.5320
Phase reg. method	ICDD (PDF 4+-2023)
Molar ratio (%)	100

3S. EPMA (electron-probe microanalysis) results

Table S14. List of constituent elements detected, and nominal chemical formulas and theoretical molecular weights calculated, from the EPMA carried out on the fabricated ceramics

Ceramic material	Constituent element	Theoretical molecular weight (g mol^{-1})	Nominal chemical formula
PT 1-P	Pb, Sr, Ti, Zr, O	321.13 ± 4.08	$(Pb_{0.93\pm0.02}Sr_{0.09\pm0.005})(Ti_{0.47\pm0.01}Zr_{0.53\pm0.01})O_{3.03\pm0.02}$
PT 2-P	Pb, Sr, Mg, Nb, Ti, Zr. Ni, O	311.17 ± 3.87	$(Pb_{0.94\pm0.02})(Sr_{0.04\pm0.005}Mg_{0.07\pm0.005}Nb_{0.24\pm0.005}Ti_{0.37\pm0.005}Zr_{0.25\pm0.005}Ni_{0.03\pm0.005})O_{2.91\pm0.02}$
PT 3-P	Pb, Mg, Nb, Ti, O	317.49 ± 2.63	$(Pb_{1.00\pm0.005})(Mg_{0.16\pm0.01}Nb_{0.45\pm0.03}Ti_{0.35\pm0.02})O_{2.99\pm0.04}$
BT 1-P	Ba, Ca, Ti, Zr, O	215.39 ± 2.54	$(Ba_{0.80\pm0.02}Ca_{0.14\pm0.005})(Ti_{0.90\pm0.005}Zr_{0.10\pm0.005})O_{2.94\pm0.02}$
BT 2-P	Ba, Sn, Ti, O	229.45 ± 1.47	$(Ba_{0.95\pm0.01})(Ti_{0.94\pm0.005}Sn_{0.06\pm0.005})O_{2.95\pm0.01}$
BT 3-P	Ba, Ca, Ti, Zr, O	214.73 ± 2.31	$(Ba_{0.79\pm0.01}Ca_{0.14\pm0.005})(Ti_{0.88\pm0.005}Zr_{0.12\pm0.005})O_{2.94\pm0.02}$

4S. Microstructure of upside-down composites

The microstructure of the BT 2-C composite is provided in Figure 2. FESEM (field-emission scanning electron microscope) micrographs and EDS (energy dispersive X-ray spectroscopy) maps for PT 1-C, PT 2-C, PT 3-C, BT 1-C, and BT 3-C are shown in Figures S3-S7 below, respectively. Area fractions of filler and binder obtained from FESEM micrographs of PT 2-C using ImageJ software for the longitudinal, lateral and top surfaces are shown in Table S15.

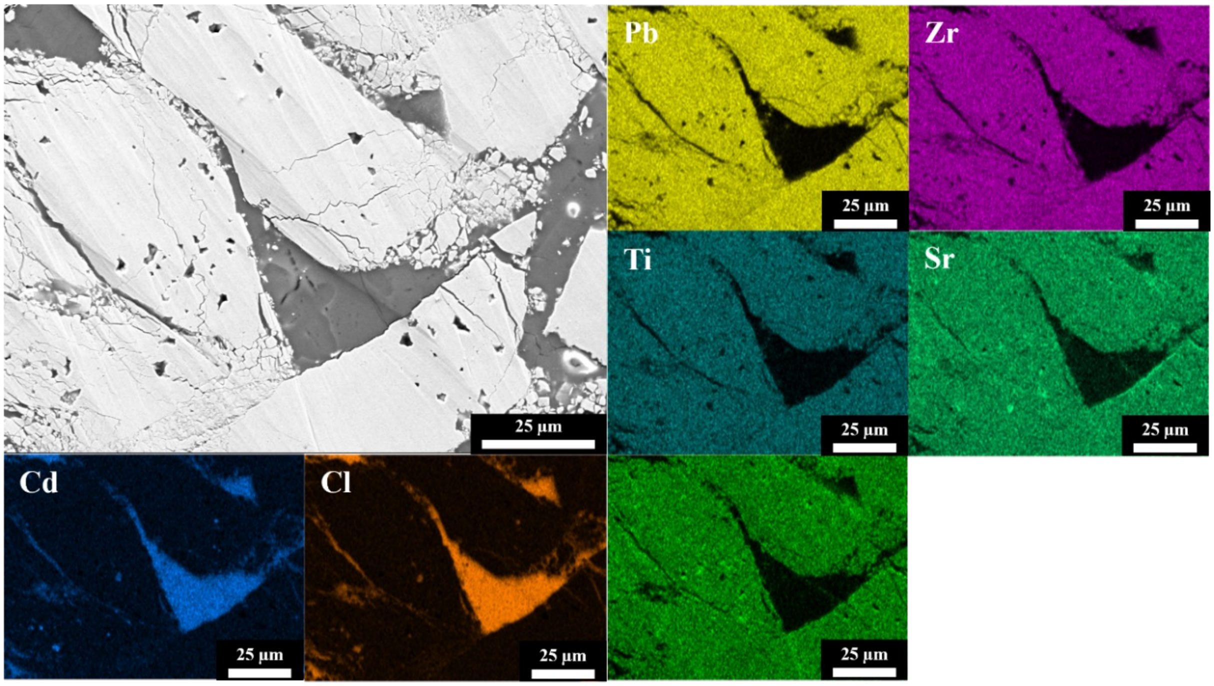

Figure S3. FESEM micrograph and EDS maps of sample PT 1-C.

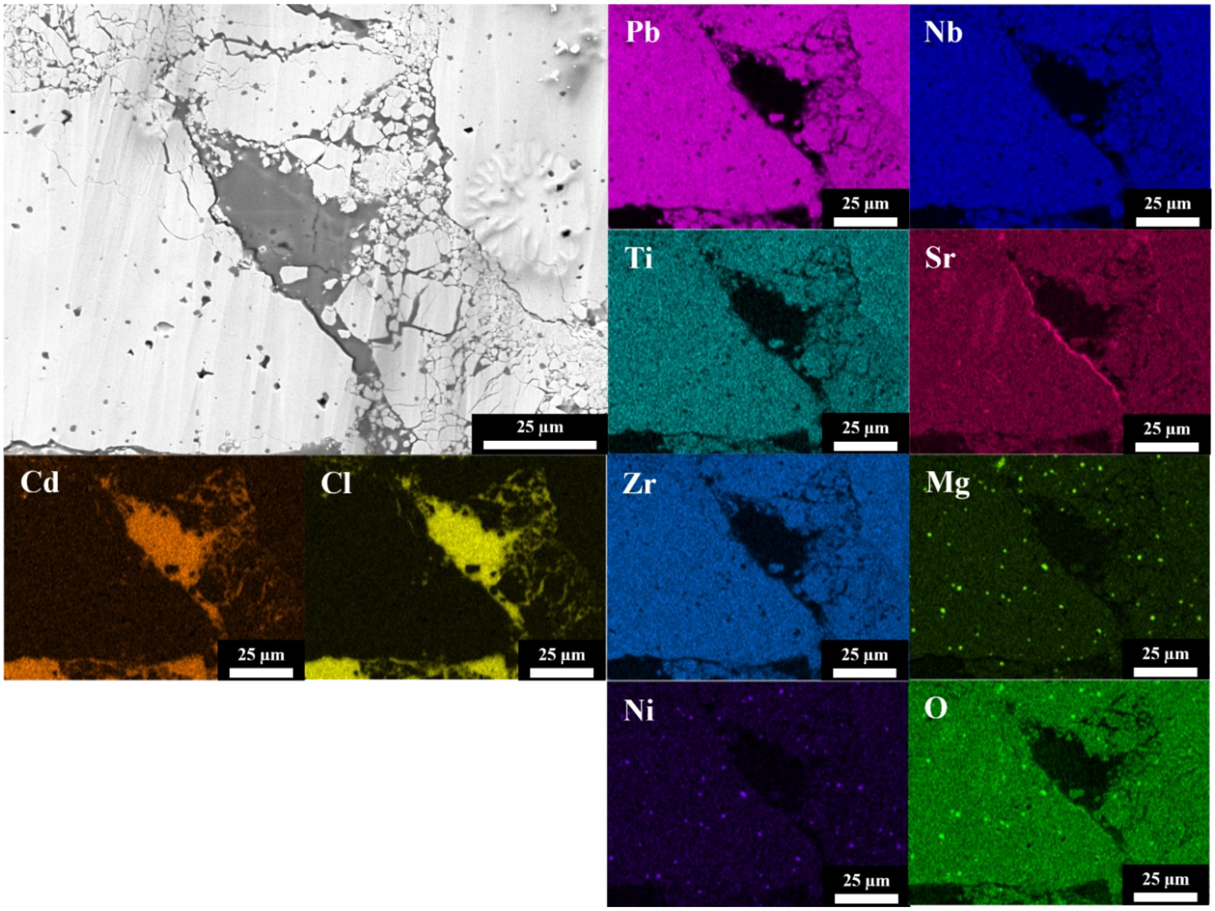

Figure S4. FESEM micrograph and EDS maps of sample PT 2-C.

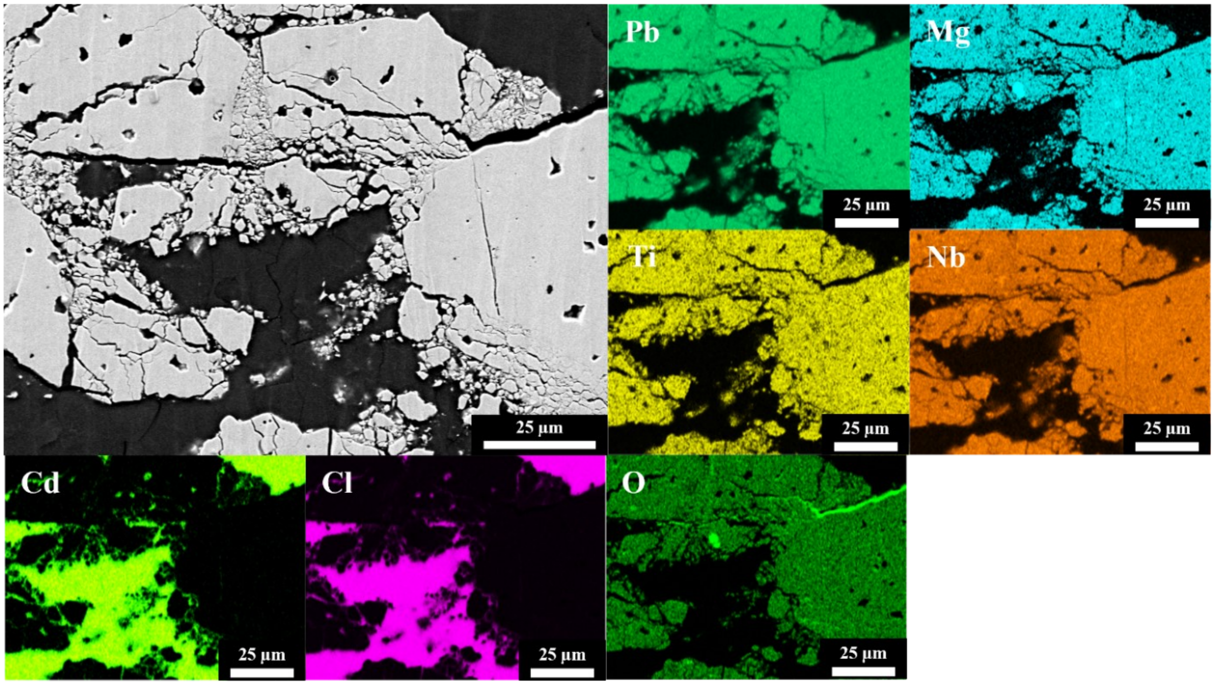

Figure S5. FESEM micrograph and EDS maps of sample PT 3-C.

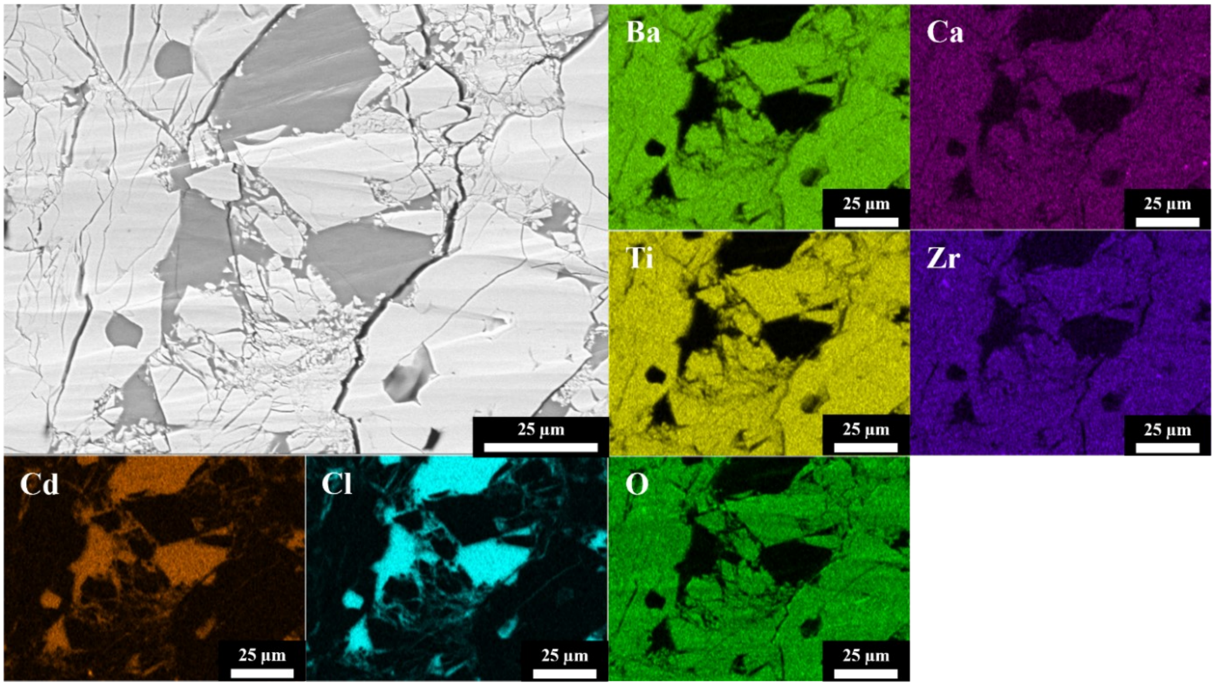

Figure S6. FESEM micrograph and EDS maps of sample BT 1-C.

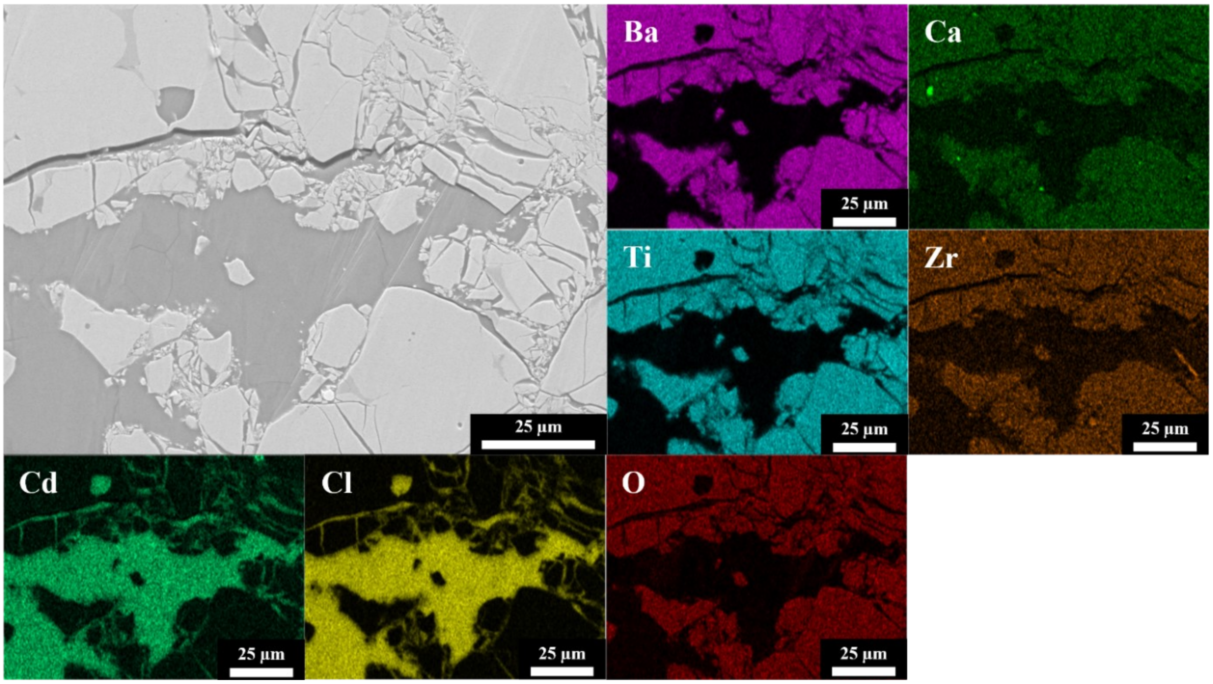

Figure S7. FESEM micrograph and EDS maps of sample BT 3-C.

Table S15. Area fractions of filler and binder obtained from FESEM micrographs of PT 2-C using ImageJ software for the longitudinal, lateral and top surfaces.

**Analyzed area**	**Probing location (randomly chosen)**	**Area fraction of $(PTMA)CdCl_3$ binder**	**Area fraction of PT 2 filler**
Longitudinal area	1	17.98	82.02
	2	19.13	80.87
	3	19.43	80.57
	4	20.80	79.20
	5	18.03	81.97
	6	18.76	81.24
Lateral area	1	19.12	80.88
	2	19.61	80.39
	3	19.45	80.55
	4	18.73	81.27
	5	19.60	80.40
	6	19.70	80.30
Surface area	1	18.43	81.57
	2	19.38	80.62
	3	17.51	82.49
	4	20.17	79.83
	5	19.83	80.18
	6	19.78	80.22

5S. Frequency dispersion of dielectric properties for the ceramics and upside-down composites

Figures S8-S13 depict the evolution of relative permittivity (ε_r) and dielectric loss (*tan δ*) from 20 Hz to 100 kHz at the unpoled and poled states for all the fabricated samples. It is noted that unlike the ceramic samples which had variations of permittivity between unpoled and poled states influenced by their respective phase structures, the corresponding composite permittivity values follow the same trend for all samples, i.e., an increase of permittivity after poling, even though the identical ceramics were present as fillers in them. Two notable contributions to this observation are the interfacial/space-charge polarizations generated and the ferroelastic nature of the binders in these composites.

In composites, the differing permittivity and conductivities between the filler and binder generate interfacial polarization according to the Maxwell-Wagner-Sillers theory.[1,2] Therefore, an electrical bilayer consisting of oppositely charged dipoles occurs at the interfaces between the fillers and the (PTMA)$CdCl_3$ binder (PTMA: $C_6H_5N(CH_3)_3$),[3] and between the composite and electrodes, which effectively contributes to the dielectric properties. The greater the mismatch of these properties, the larger the contribution from the interfacial polarization, which is supported by the trend of the average values of ε_c measured for the Pb-based and Pb-free sets of the composites (see Figure 3d and 4d) compared to the corresponding ε_p (see Figure 3a and 4a), respectively. From these observations, it can be deduced that upon poling of the fillers, additional interfacial polarization is induced from the spontaneous ferroelectric polarization generated from the filler inclusions through electrostatic interactions, which was effectively zero before poling, when the domains inside the filler were randomly oriented. Additionally, this induced polarization is isotropic in nature, i.e., the polarization is generated uniformly across the bilayers and is, therefore, independent of the phase structure of the filler which effectively manifests as an increase in the permittivity after poling. Both poled and unpoled permittivity of all the composite samples exhibit a decreasing trend with frequency (see Figures S8-S13), as the interfacial polarization is frequency-dependent and cannot respond to faster field switching.

Another notable contribution to the dielectric properties is the ferroelastic nature of the (PTMA)$CdCl_3$ binder,[3,4] which can ease the strain generated by the domain wall pinning effect of the fillers during poling. As the binder was able to shift its orientation state based on the stress generated by the fillers after poling, the charge transfer between lattices could be moderated better

as opposed to the situation before poling. This would decrease the dielectric loss after poling as seen in Figures S8-S13, which would also consequently be an added contribution to the real part of permittivity.

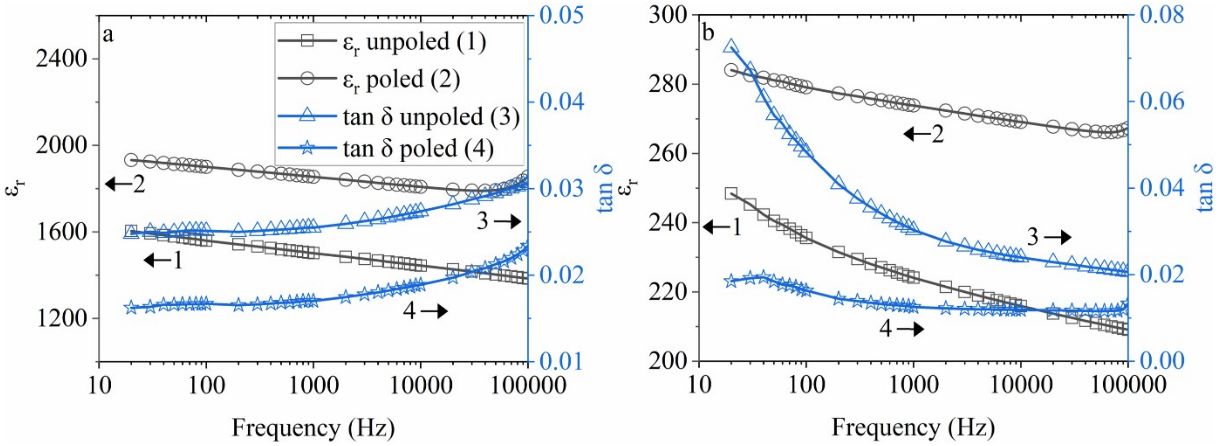

Figure S8. Dependence of ε_r and tan δ on frequency for specimens of the (a) PT 1-P and (b) PT 1-C samples measured at the unpoled and poled states.

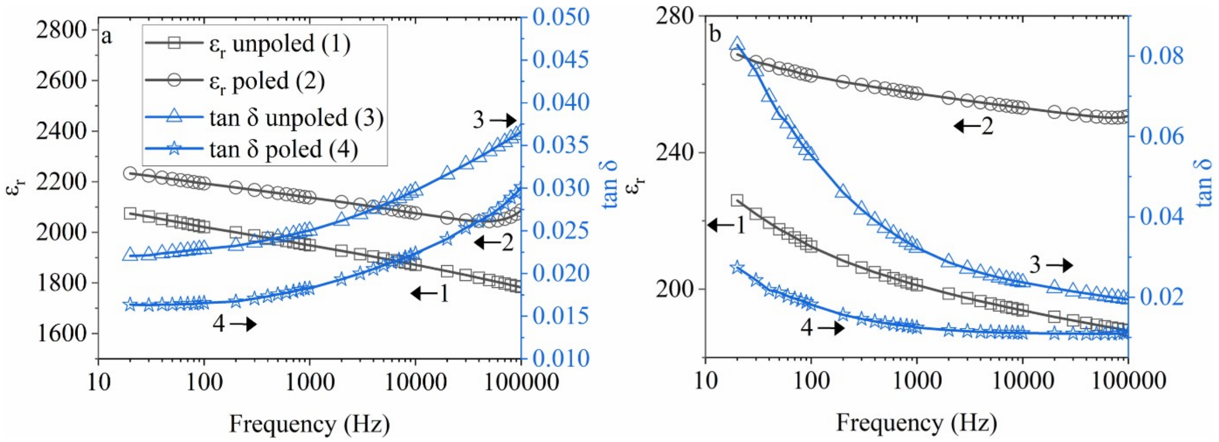

Figure S9. Dependence of ε_r and tan δ on frequency for specimens of the (a) PT 2-P and (b) PT 2-C samples measured at the unpoled and poled states.

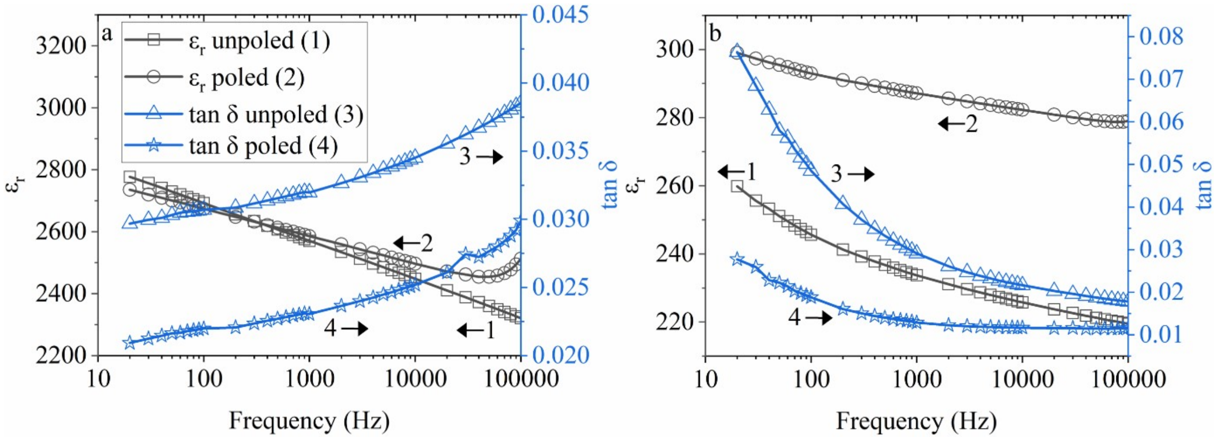

Figure S10. Dependence of ε_r and tan δ on frequency for specimens of the (a) PT 3-P and (b) PT 3-C samples measured at the unpoled and poled states.

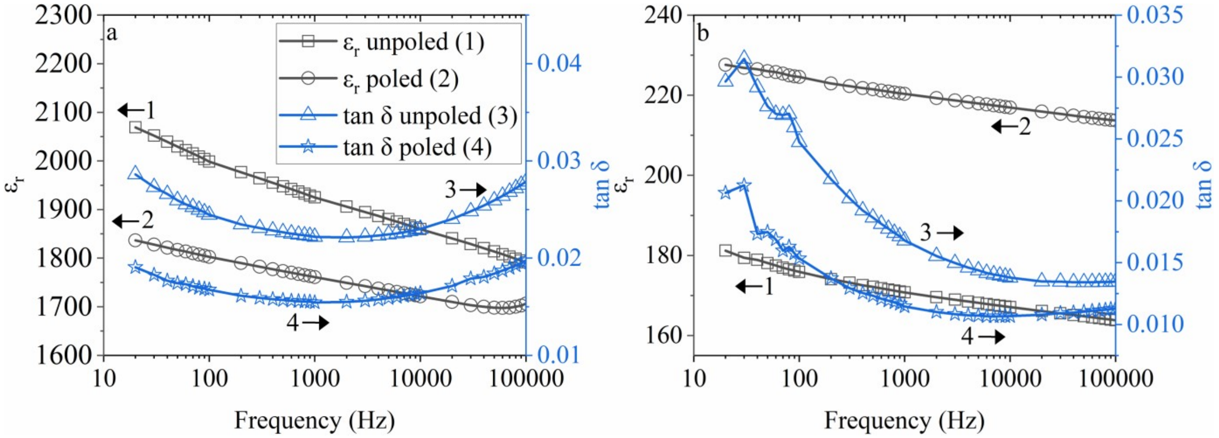

Figure S11. Dependence of ε_r and tan δ on frequency for specimens of the (a) BT 1-P and (b) BT 1-C samples measured at the unpoled and poled states.

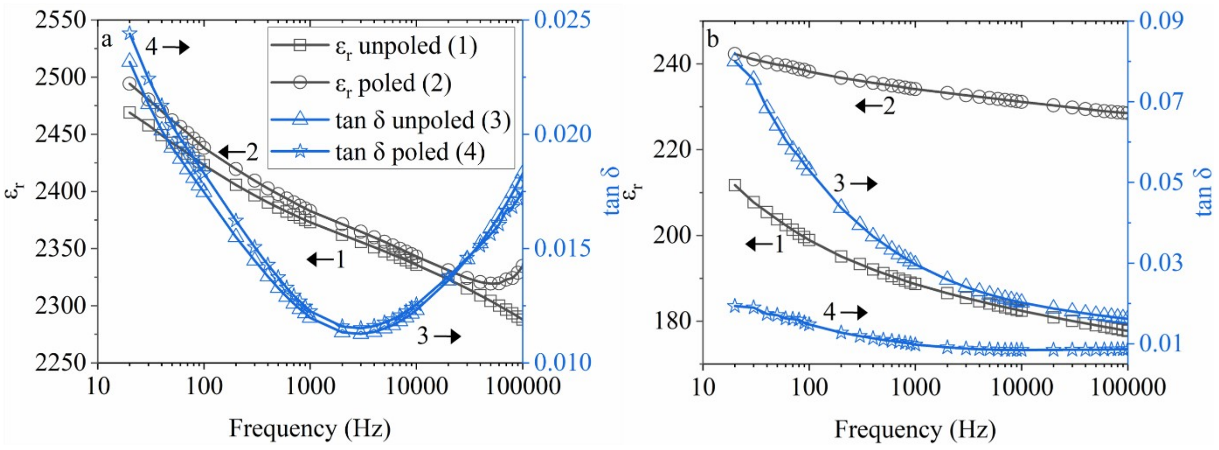

Figure S12. Dependence of ε_r and tan δ on frequency for specimens of the (a) BT 2-P and (b) BT 2-C samples measured at the unpoled and poled states.

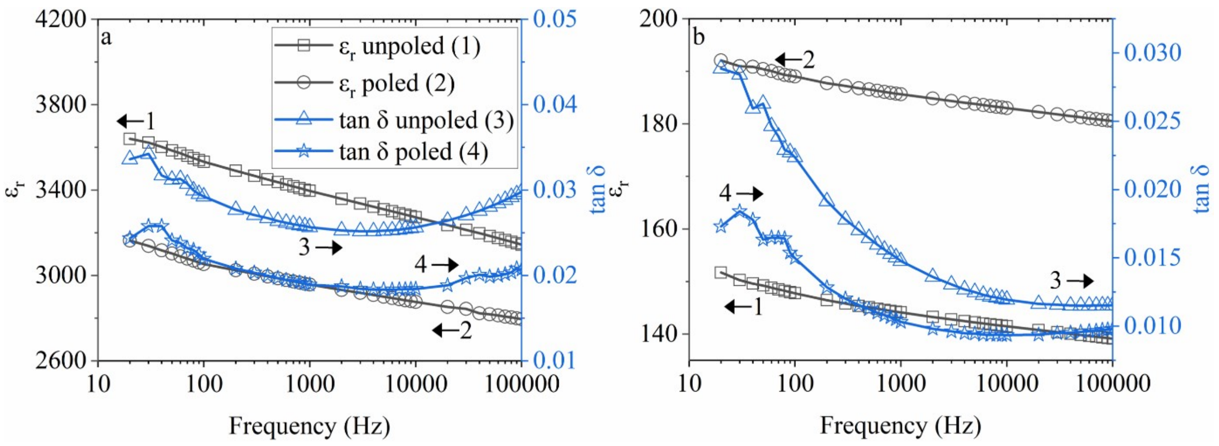

Figure S13. Dependence of ε_r and tan δ on frequency for specimens of the (a) BT 3-P and (b) BT 3-C samples measured at the unpoled and poled states.

6S. Dielectric mechanisms in upside-down composites

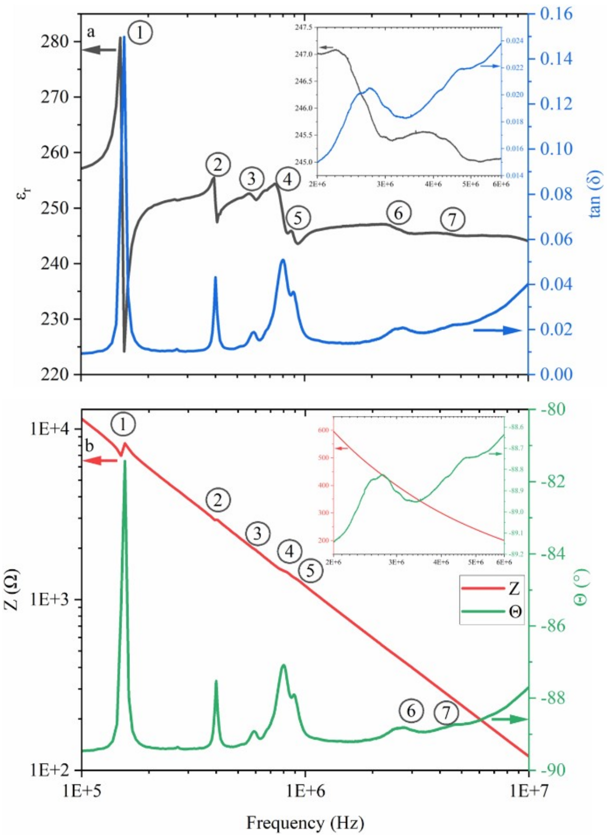

Figure S14. Dependence of (a) ε_r and *tan δ* and (b) impedance (*Z*) and phase angle (*Θ*) on frequency for PT 2-C in the poled state measured with impedance analyzer. Relaxations and resonance dispersions are marked with numbers.

7S. Piezoelectric properties from impedance analysis

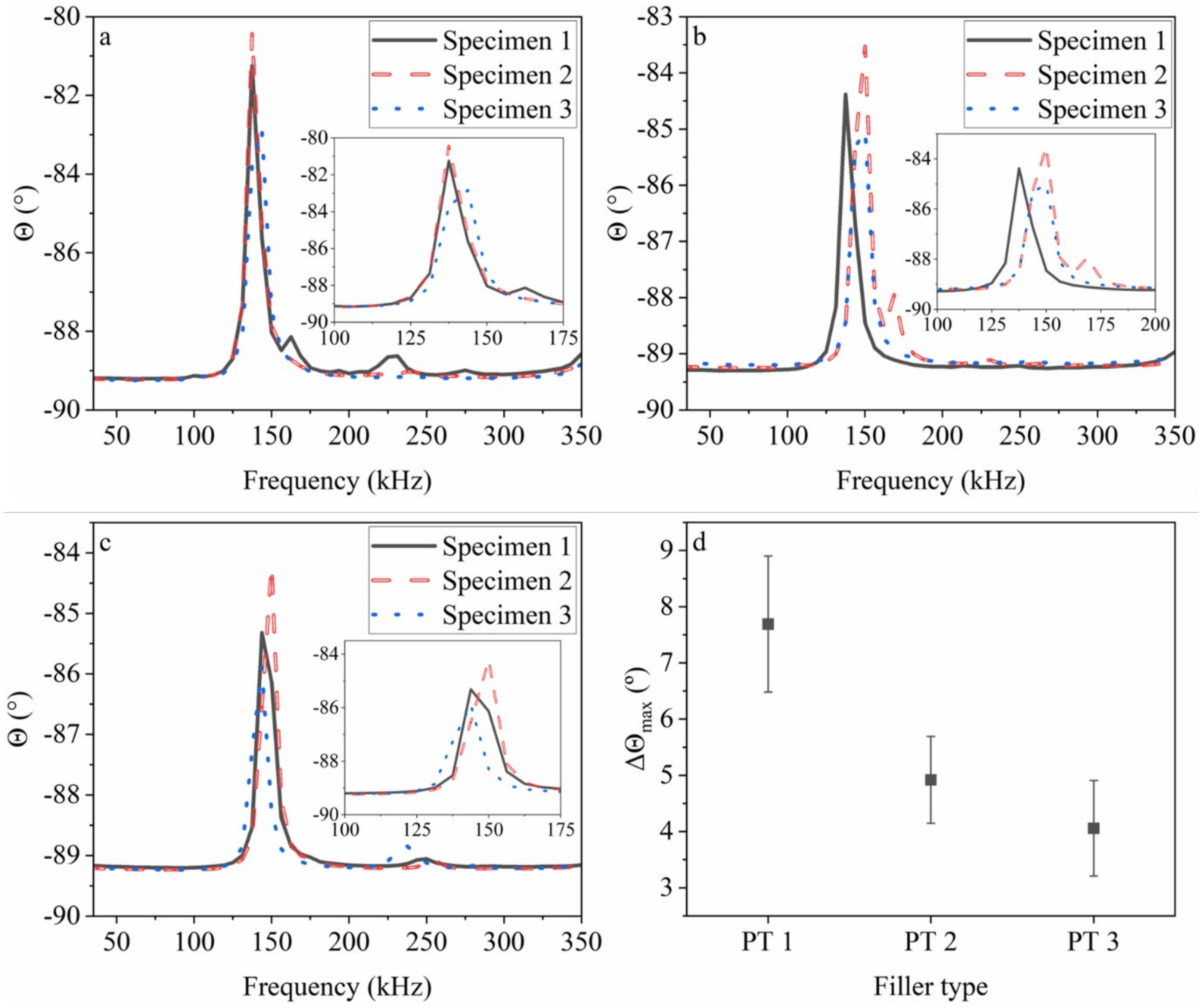

Figure S15. Dependence of Θ on frequency for the poled (a) PT 1-C, (b) PT 2-C and (c) PT 3-C composite samples, and (d) comparison of the phase angle difference between the baseline and the first harmonic ($\Delta\Theta_{max}$) for these samples.

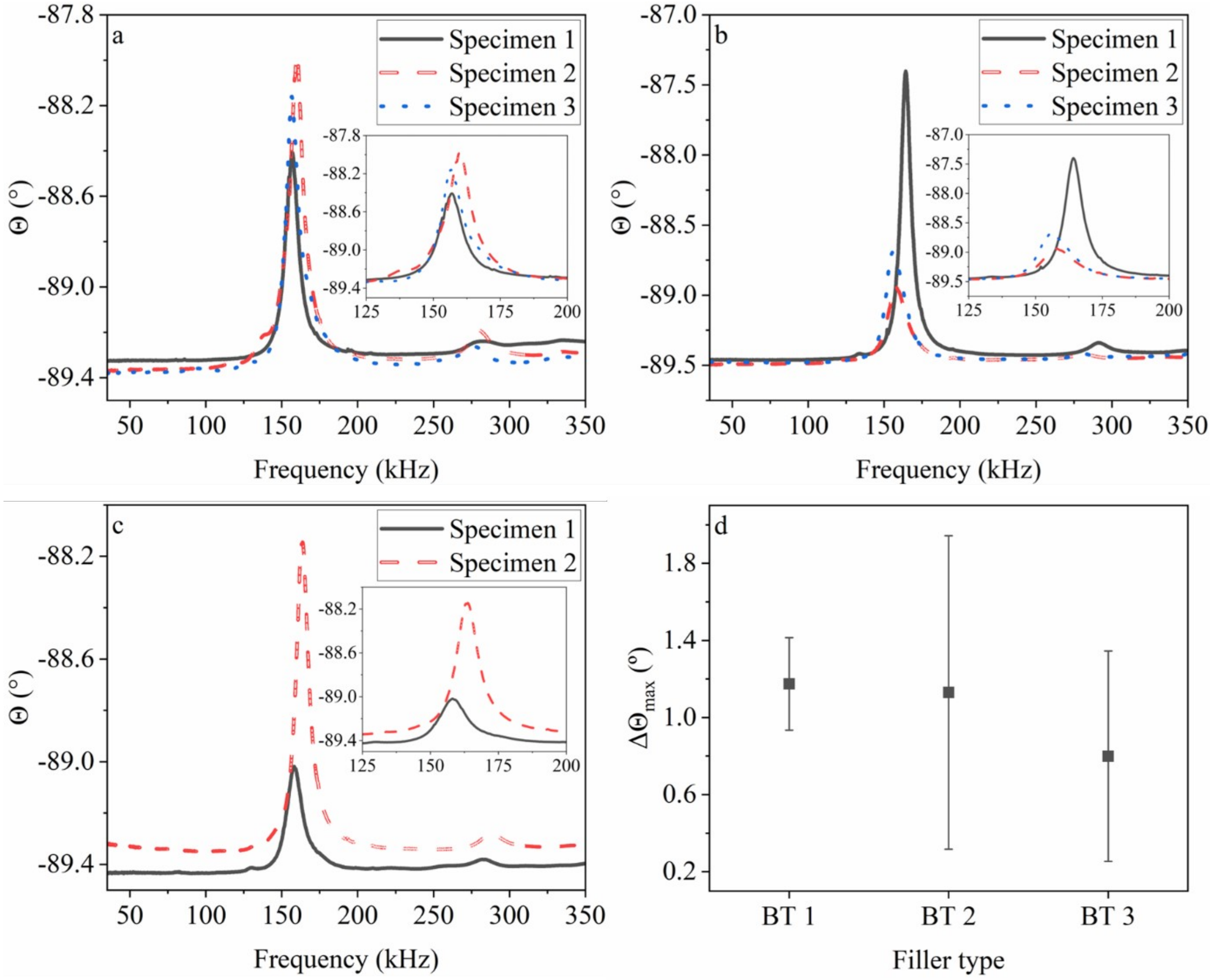

Figure S16. Dependence of Θ on frequency for the poled (a) BT 1-C, (b) BT 2-C and (c) BT 3-C composite samples, and (d) comparison of $\Delta\Theta_{\max}$ for these samples.

8S. Description of the electric field on the fillers of upside-down composites

8S.1 Analytical equation for electric field on the fillers

Based on the connectivity of the fillers, different responses of composites towards a dynamic applied electric field have been suggested in literature[5–7] by considering the transient development of local electric field in the phases of the composite. The upside-down composites fabricated in this work have a 0-3 connectivity between the filler and binder respectively.[8, 9] The microstructure consisted of discrete fillers which are surrounded completely by a three-dimensionally connected binder. This can be validated by the FESEM micrographs of the composites displayed in Figure 2 and Figures S3-S7, and also by the fabrication methodology where we consolidate discrete/crushed recycled ceramic filler particles under pressure with a suitable binder that fills the interfaces due to capillary pressures and forms a densified bridge between them upon crystallization.[10]

For 0-3 composites, the local electric field in the fillers based on the applied external electric field is given in literature[5] by Equation S1.

$$\frac{\partial\langle E_f\rangle}{\partial t}+\frac{\langle E_f\rangle}{\tau}=\frac{3\left[\sigma_b\langle E_{app}\rangle+\frac{\varepsilon_b\partial\langle E_{app}\rangle}{\partial t}\right]-(1-\varphi_f)\frac{\partial\langle P_f\rangle}{\partial t}}{3\varphi_f\varepsilon'_b+(1-\varphi_f)(\varepsilon'_f+2\varepsilon'_b)}$$

(S1)

The angular brackets (⟨⟩) indicate a volume-averaged parameter, E_{app} is the applied electric field, E is the electric field, P is the polarization, σ is the conductivity, ε' is the permittivity of a particular phase, τ is the relaxation time of the interfacial polarization generated in the composite, and the other quantities and relevant subscripts are identical to Equations 4-5. Equation S2 then provides the analytical equation of τ.

$$\tau=\frac{3\varphi_f\varepsilon'_b+(1-\varphi_f)(\varepsilon'_f+2\varepsilon'_b)}{3\varphi_f\sigma_b+(1-\varphi_f)(\sigma_f+2\sigma_b)}$$

(S2)

The conductivities measured with the source meter and the permittivity data for the Pb-based ceramics used as fillers and the $(PTMA)CdCl_3$ binder are given in Table S16. The corresponding

values of relaxation times of the interfacial polarization calculated for the Pb-based upside-down composites are given in Table S17.

Table S16. Conductivity and permittivity data for the Pb-based ceramics and $(PTMA)CdCl_3$ binder

Type of sample	σ (S/m)	ε (F/m)
$(PTMA)CdCl_3$	8.931E-13	4.43E-11
PT 1-P	2.160E-10	1.33E-08
PT 2-P	2.753E-10	1.73E-08
PT 3-P	5.880E-10	2.28E-08

Table S17. Relaxation times of the interfacial polarization calculated for the Pb-based composites from Equation S2

Type of sample	τ (s)
PT 1-C	60.85
PT 2-C	62.07
PT 3-C	38.95

Based on the relevant parameters from Tables S16-S17, the equation for transient electric field on the fillers of each Pb-based composite is given by Equations S3-S5 for PT 1-C, PT 2-C and PT 3-C, respectively.

$$\frac{\partial\langle E_f\rangle}{\partial t} + 0.164\langle E_f\rangle = 0.0508\left[\langle E_{app}\rangle + \frac{\partial\langle E_{app}\rangle}{\partial t}\right] - (7.16E7)\frac{\partial\langle P_f\rangle}{\partial t} \quad \text{(S3)}$$

$$\frac{\partial\langle E_f\rangle}{\partial t} + 0.161\langle E_f\rangle = 0.0395\left[\langle E_{app}\rangle + \frac{\partial\langle E_{app}\rangle}{\partial t}\right] - (5.56E7)\frac{\partial\langle P_f\rangle}{\partial t} \quad \text{(S4)}$$

$$\frac{\partial\langle E_f\rangle}{\partial t} + 0.257\langle E_f\rangle = 0.0303\left[\langle E_{app}\rangle + \frac{\partial\langle E_{app}\rangle}{\partial t}\right] - (4.26E7)\frac{\partial\langle P_f\rangle}{\partial t} \quad \text{(S5)}$$

Using the fillers' ferroelectric data which provide the variation of polarization with time, exact solutions of $\langle E_f\rangle$ can be obtained under various time periods. However, this is beyond the scope of this work.

When small electric fields are applied, for instance, during the acquisition of the dielectric data from the LCR meter (≈ 1 V) or when the effect of interfacial polarization is not considered, i.e., $\frac{\partial\langle P_f\rangle}{\partial t} = 0$, Equation S1 can be simplified to Equation S6.

$$\frac{\langle E_f\rangle}{\langle E_{app}\rangle} = \begin{cases} \frac{3\varepsilon'_b}{(1-\varphi_f)\varepsilon'_f + (2+\varphi_f)\varepsilon'_b}, & t \ll \tau \\ \frac{3\sigma_b}{(1-\varphi_f)\sigma_f + (2+\varphi_f)\sigma_b}, & t \gg \tau \end{cases} \quad \text{(S6)}$$

$\frac{\langle E_f\rangle}{\langle E_{app}\rangle}$ represents the electric field on the fillers as a fraction of the applied external electric field. Compared to the values of τ calculated for each composite in Table S17, the largest time period between each pulse of the externally applied electric field during the LCR measurement, i.e., 20 Hz, is much smaller (0.05 s), which satisfies the first condition. This meant that the fraction of electric field on the fillers depended upon the ratio of ε'_b to ε'_f or ε_b to ε_f, which is concurrent with the experimental results obtained in Section 3.3.

The situation becomes more complex during the poling procedure where a larger electric field is applied (3 kV mm^{-1}) and the effect of interfacial polarization is assumed to be significant since it is this polarization that poles the ferroelectric fillers. As our poling time (10 minutes) is relatively larger than the values of τ in Table S17 for all composites, and since we used a direct current (DC) poling field which has no alternation in the electric field direction, it can be assumed that the interfacial polarization generated at the interfaces is relatively equal in all three composites. In this case, the d_{33} should have been saturated in all three composites contrary to the trend observed in experiments where the saturation is only observed for PT 2-C and PT 3-C but not for PT 1-C.

We attribute this reason to a relaxation time of interfacial polarization that is actually much larger than the values calculated in Table S17 for the PT 2-C and PT 3-C. Owing to a universal 'clustering effect' that occurs in all the three composites due to the very high filler volume fractions, the electric field in the numerous amount of neck regions present between closely contacting fillers is large, as confirmed by finite elemental methods in previous work.[11] In this special case, the relaxation times might be less dependent on the conductivity of the fillers and more on that of the binder. As the binder is the same for all the composites considered, the relaxation times depend only on the permittivity difference between the fillers from Equation S2.

For clarifying this phenomenon, the PT 1-C and PT 2-C were poled with a longer poling time (36 minutes) in our other work.[12] The percentage change in d_{33} obtained for the PT 1-C and PT 2-C by using the poling time of this work and the longer poling time was 0.89 % and 34.5 %, respectively. This is because the τ for the PT 2-C was far higher than the calculated one in Table S17, which means that the effect of interfacial polarization for the PT 2-C and the PT 3-C can be neglected, i.e., $\frac{\partial \langle P_f \rangle}{\partial t} = 0$. Therefore, the condition of $t \ll \tau$ would be valid specifically for these composites and the fraction of electric field on the fillers depended upon the ratio of ε_b to ε_f which is concurrent with the experimental results obtained in Section 3.3 and is similar to the case of LCR measurement. As for the PT 1-C, although the filler volume fractions are large, like for the PT 2-C and PT 3-C, the shorter relaxation time owing to a lower filler permittivity enables a fully poled condition even under short poling times. Thus, the electric field permeation is theorized to reach a percolation threshold owing to the large contribution of $\frac{\partial \langle P_f \rangle}{\partial t}$ which makes Equation S6 inapplicable for this sample. Nevertheless, Equations S3-S5 are still useful to describe the trends of the electric field on the fillers of upside-down composites with varying permittivity values.

As it is established that the permittivity variation of the fillers drastically influences the electric field on the fillers in the cases of poling as well as the LCR measurement, the following section investigates the physical mechanism behind the saturation phenomenon of the electrical properties observed in these composites.

8S.2 Physical mechanism for the saturation phenomenon of electrical properties in upside-down composites

As the electric field on the active filler phase in the composites varies based on the disparateness between permittivity values of the filler and binder based on the discussion in Section 8S.1, the electric field distribution across the composite proves to be erratic based on the location in the composite.[13, 14] This can be modelled as capacitor-like formations[11] on each filler-binder interface that forces the amount of charge stored which is proportional to the electric field across it to be determined by the dielectric permittivity difference between the filler (dielectric medium) and the surrounding binder (external environment) or *vice-versa*, as shown in Figure S17. For instance, in a binder-filler-binder interface, the dielectric medium being the filler possesses a large permittivity, i.e., a larger capacitance compared to a neighboring filler-binder-filler interface, where the dielectric medium is the low permittivity binder. This produces an equivalent circuit of capacitors alternating in high and low capacitances across the entire composite and therefore, the electric field distribution follows the opposite trend of these capacitances by Kirchoff's laws.

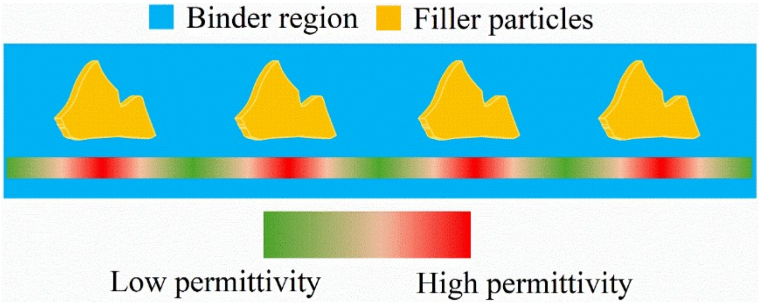

Figure S17. Schematic of capacitor-like formations at filler-binder interfaces of upside-down composites.

Thus, in a highly simplified and volume-averaged view, the composite can be estimated as two capacitors connected in series representing each phase (C_f and C_b) where their respective capacitances are governed by the permittivity of each phase, as shown in Figure S18a. The electric field in the binder (V_b) can then be calculated based on the applied field (V_{app}) according to Equation S7. The composite's capacitance (C_c) representing ε_c can then be computed based on

these individual capacitances through Equation S8, which then produces an equivalent circuit shown in Figure S18b.

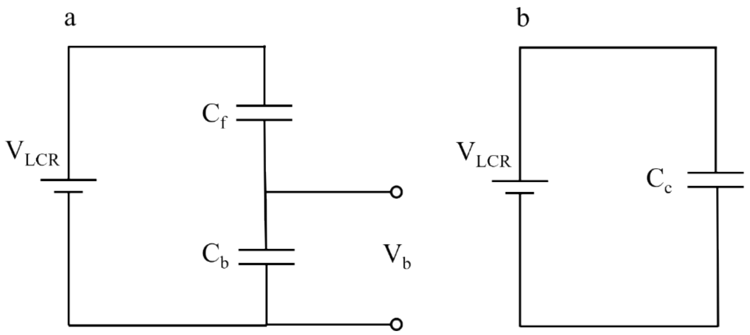

Figure S18. Simplified equivalent circuits for piezoelectric composites consisting of (a) capacitors connected in series and (b) one capacitor with a summed-up capacitance from (a).

$$V_b = \frac{V_{app} C_f}{C_f + C_b} \tag{S7}$$

$$C_c = \frac{C_b C_f}{C_f + C_b} \tag{S8}$$

From Equations S7-S8, it can be easily identified that for a constant value of V_{app} and C_b, as the C_f increases, the V_b and C_c initially increase and saturate over very large values of C_f. This points to the domination of electric field by the binder over very large ε_f values and the decreased contribution of ε_f towards ε_c, respectively. Eventual complete negation of the electric field on the filler with the increase of ε_f is attributed to being the physical mechanism of the saturating effects of permittivity and d_{33c}, hindering the charge migration to and from the interfaces between the filler and binder.

The saturation effect has also been confirmed via finite elemental method for conventional piezoelectric composites[11] and represents the asymptotic part of the trends of ε_c in Figure 5a, for both sets of composites. At lower values of C_f, although the absolute value of C_c is low, the contribution of C_f is higher owing to the more homogenous electric field distribution,[11] and represents the non-linear increasing part of the trends of ε_c in Figure 5a, for both sets of composites.

9S. Comparison of different modelling approaches

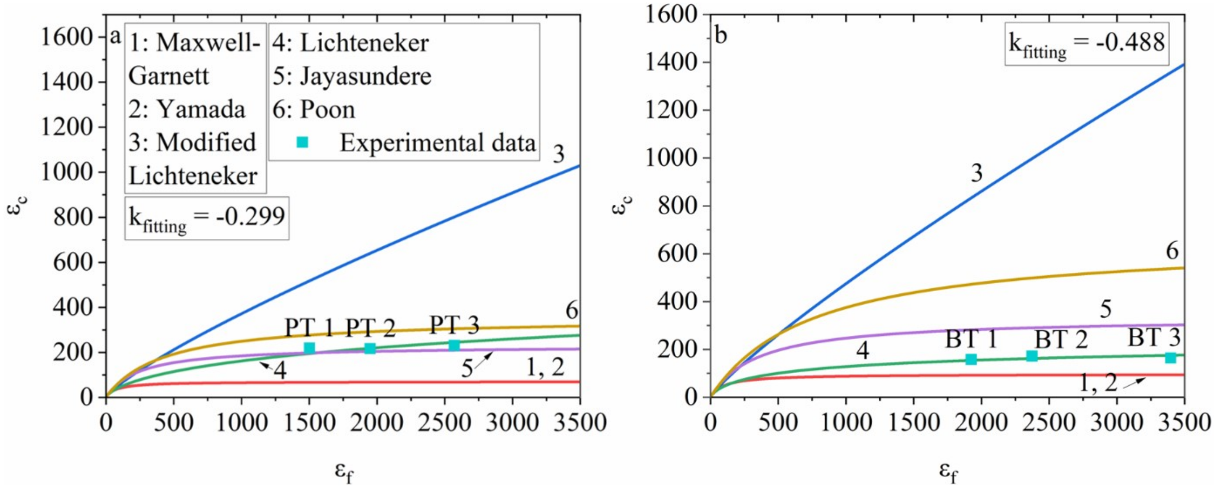

Figure S19. Dependence of composite relative permittivity (ε_c) on filler relative permittivity (ε_f) derived from different models and obtained from experimental data for the (a) Pb-based and (b) Pb-free composite samples. $k_{fitting}$ is the value of the fitting constant (k) used for the best fitting of the Lichteneker model to the experimental datapoints obtained via the Least-Squares method.

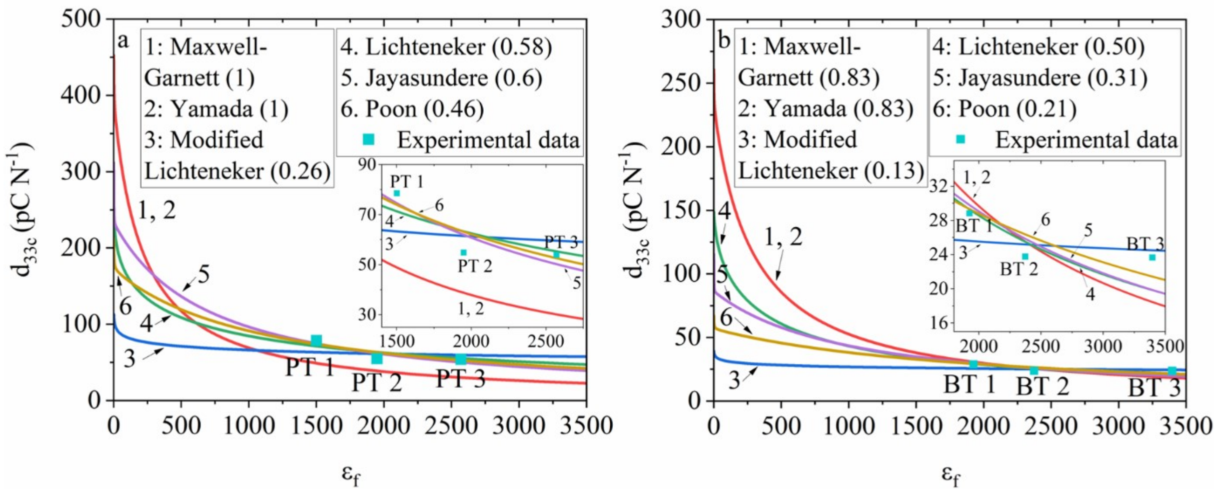

Figure S20. Dependence of composite d_{33} (d_{33c}) on ε_f derived from different models using the results in Figure S17 and obtained from experimental data for the (a) Pb-based and (b) Pb-free composite samples. The values of $\alpha_{fitting}$ used for each model are also given in the brackets, where $\alpha_{fitting}$ is the poling efficiency (α) used for the best fitting of each model to the experimental datapoints obtained via the Least-Squares method. The inverse depolarization factor (n) is fixed to a constant of 3.

Table S18. Errors of fitting results against experimental ε_c and d_{33c} determined via the Least-Squares method

**Type of sample**	**Model**	**ε_c error**	**d_{33c} error**
Pb-based	Maxwell-Garnett	71792	1701
	Yamada	71792	1701
	Modified Lichteneker	593521	309
	Lichteneker	789	126
	Jayasundere	1076	80
	Poon	14358	93
Pb-free	Maxwell-Garnett	15537	34
	Yamada	15537	34
	Modified Lichteneker	2561809	13
	Lichteneker	232	18
	Jayasundere	48464	20
	Poon	345095	12

10S. Extension of modelling approach to other fillers

Table S19. Compositional, dielectric and piezoelectric data of piezoceramics extracted from literature

Reference datapoint name	Composition	d_{33p} (pC N^{-1})	Unpoled ε_p	Reference
R-1	$0.96(K_{0.48}Na_{0.52})(Nb_{0.95}Sb_{0.05})O_3$-$0.04Bi(Na_{0.41}K_{0.09}Zr_{0.5})O_3$	490	2300	15
R-2	$0.9575(K_{0.48}Na_{0.52})(Nb_{0.95}Sb_{0.05})O_3$-$0.0425(Bi_{0.5}Ag_{0.5})ZrO_3$	490	3167	16
R-3	$(Ba_{0.85}Ca_{0.15})(Ti_{0.9}Sn_{0.1})O_3$ + 0.3 wt.% Li_2CO_3	485	4790	17
R-4	$(Ba_{0.85}Ca_{0.15})(Ti_{0.9}Zr_{0.1})O_3$ + 0.5 wt.% Li_2CO_3	493	5289	18
R-5	$0.97[0.67Bi_{1.05}FeO_3$-$0.33BaTiO_3]$-$0.03Bi_{1.05}(Zn_{0.5}Ti_{0.5})O_3$	324	500	19
R-6	$0.95(Na_{0.49}K_{0.49}Li_{0.02})(Nb_{0.8}Ta_{0.2})O_3$-$0.05CaZrO_3$ + 2 wt.% MnO_2	320	1625	20
R-7	$(Na_{0.52}K_{0.4375})(Nb_{0.8875}Sb_{0.07})O_3$–$0.0425LiTaO_3$	330	2100	21
R-8	$(Ba_{0.94}Ca_{0.06})(Ti_{0.95}Sn_{0.05})O_3$	325	3060	22

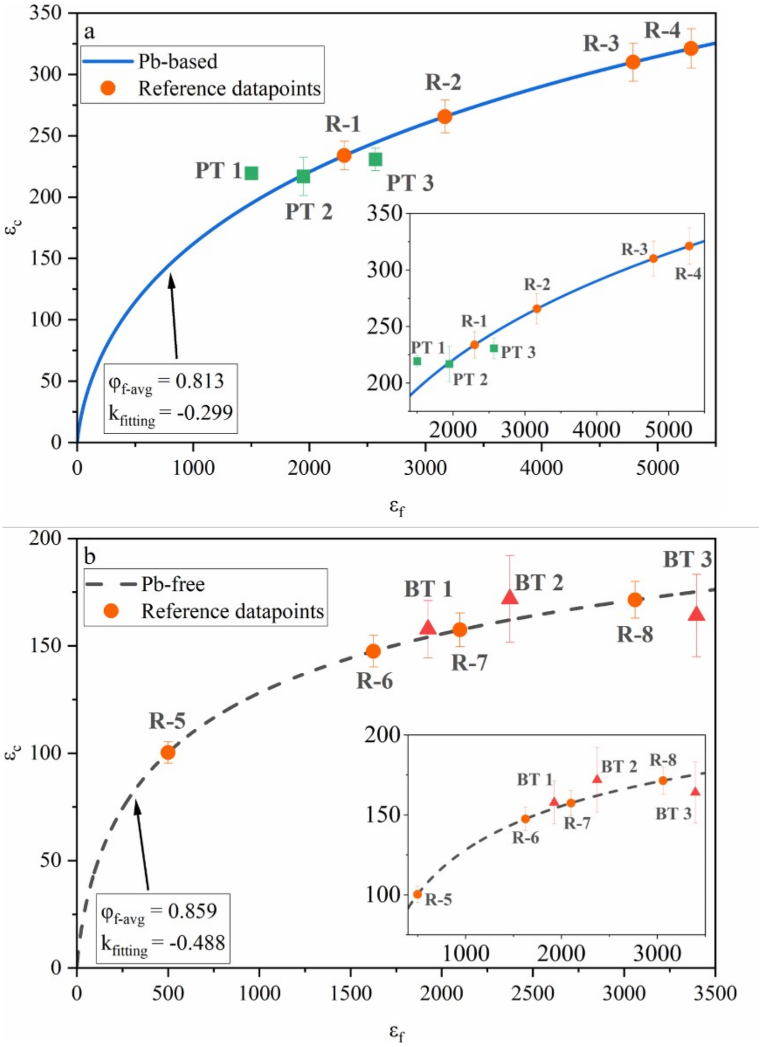

Figure S21. Dependence of unpoled ε_c on ε_f for (a) Pb-based fillers of this work and R-1−R-4 from literature[15–18], and (b) Pb-free fillers of this work and R-5−R-8 from literature.[19–22]

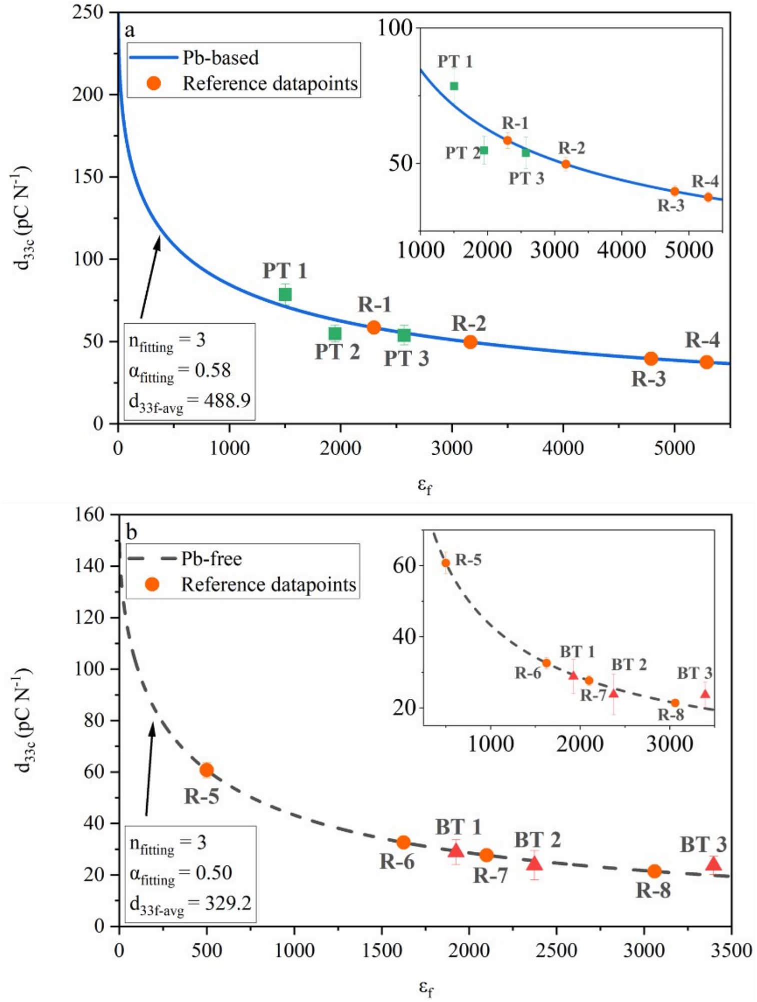

Figure S22. Dependence of unpoled d_{33c} on ε_f for (a) Pb-based fillers of this work and R-1−R-4 from literature[15–18], and (b) Pb-free fillers of this work and R-5−R-8 from literature.[19–22] The unit of $d_{33f\text{-}avg}$ is pC N^{-1}.

References

1. Tian G, Deng W, Yang T, Xiong D, Zhang H, Lan B, et al. Insight into Interfacial Polarization for Enhancing Piezoelectricity in Ferroelectric Nanocomposites. Small. 2023; 19(16): 2207947. https://doi.org/10.1002/smll.202207947
2. Lau ST, Kwok KW, Shin FG, Kopf S. A poling study of lead zirconate titanatepolyurethane 0-3 composites. J Appl Phys. 2007; 102(4): 044104. https://doi.org/10.1063/1.2767614
3. Hu Y, You L, Xu B, Li T, Morris SA, Li Y, et al. Ferroelastic-switching-driven large shear strain and piezoelectricity in a hybrid ferroelectric. Nat Mater. 2021; 20: 612–617. https://doi.org/10.1038/s41563-020-00875-3
4. Tabeshfar M, Nelo M, Anandakrishnan SS, Siddiqui M, Peräntie J, Tofel P, et al. Oxide-halide perovskite composites for simultaneous recycling of lead zirconate titanate piezoceramics and methylammonium lead iodide solar cells. Small Methods. 2023; 8(5): 2300830. https://doi.org/10.1002/smtd.202300830
5. Kwok KW, Lau ST, Wong CK, Shin FG. Effects of electrical conductivity on poling of ferroelectric composites. J Phys D Appl Phys. 2007; 40(21): 6818–6823. https://doi.org/10.1088/0022-3727/40/21/049
6. Kar-Gupta R, Venkatesh TA. Electromechanical response of 1-3 piezoelectric composites: Effect of poling characteristics. J Appl Phys. 2005; 98(5). 054102. https://doi.org/10.1063/1.2014933
7. Bowen CR, Perry A, Kara H, Mahon SW. Analytical modelling of 3-3 piezoelectric composites. J Eur Ceram Soc. 2001; 21(10–11): 1463–1467. https://doi.org/10.1016/S0955-2219(01)00042-5
8. Newnham RE, Skinner DP, Cross LE. Connectivity and piezoelectric-pyroelectric composites. Mater Res Bull. 1978; 13(5): 525–536. https://doi.org/https://doi.org/10.1016/0025-5408(78)90161-7
9. Holterman J, Groen P. Piezoelectric Materials. In: *An Introduction to Piezoelectric Materials and Applications*. Apeldoorn: Stichting Applied Piezo Netherlands; 2013: 98-100
10. Guo J, Floyd R, Lowum S, Maria JP, de Beauvoir TH, Seo JH, et al. Cold Sintering: Progress, Challenges, and Future Opportunities. Annu Rev Mater Res. 2019; 49: 275–295. https://doi.org/10.1146/annurev-matsci-070218-010041
11. Padurariu L, Horchidan N, Ciomaga CE, Curecheriu LP, Lukacs VA, Stirbu RS, et al.

Influence of Ferroelectric Filler Size and Clustering on the Electrical Properties of (Ag-BaTiO3)-PVDF Sub-Percolative Hybrid Composites. ACS Appl Mater Interfaces. 2023; 15(4): 5744–5759. https://doi.org/10.1021/acsami.2c15641

12. Tabeshfar M, Nelo M, Anandakrishnan SS, Peräntie J, Bai Y. Study on recycling of lead-based piezoceramics using trimethylchloromethyl ammonium-based halide perovskite binder. J Am Ceram Soc. 2025; 108: e20714. https://doi.org/10.1111/jace.20714

13. Padurariu L, Brunengo E, Canu G, Curecheriu LP, Conzatti L, Buscaglia MT, et al. Role of Microstructures in the Dielectric Properties of PVDF-Based Nanocomposites Containing High-Permittivity Fillers for Energy Storage. ACS Appl Mater Interfaces. 2023; 15(10): 13535–13544. https://doi.org/10.1021/acsami.2c23013

14. Xie Z, Liu D, Xiao Y, Wang K, Zhang Q, Wu K, et al. The effect of filler permittivity on the dielectric properties of polymer-based composites. Compos Sci Technol. 2022; 222: 109342. https://doi.org/10.1016/j.compscitech.2022.109342

15. Wang X, Wu J, Xiao D, Zhu J, Cheng X, Zheng T, et al. Giant piezoelectricity in potassium-sodium niobate lead-free ceramics. J Am Chem Soc. 2014; 136(7): 2905–2910. https://doi.org/10.1021/ja500076h

16. Wang X, Wu J, Xiao D, Cheng X, Zheng T, Lou X, et al. New potassium-sodium niobate ceramics with a giant d 33. ACS Appl Mater Interfaces. 2014; 6(9): 6177–6180. https://doi.org/10.1021/am500819v

17. Zhao L, Zhang BP, Zhou PF, Zhao XK, Zhu LF. Phase structure and property evaluation of (Ba,Ca)(Ti,Sn)O3 sintered with Li2CO3 addition at low temperature. J Am Ceram Soc. 2014; 97(7): 2164–2169. https://doi.org/10.1111/jace.12939

18. Chen X, Li Y, Zeng J, Zheng L, Park CH, Li G. Phase Transition and Large Electrostrain in Lead-Free Li-Doped (Ba, Ca)(Ti, Zr)O3 Ceramics. J Am Ceram Soc. 2016; 99(6): 2170–2174. https://doi.org/10.1111/jace.14184

19. Lee MH, Kim DJ, Park JS, Kim SW, Song TK, Kim MH, et al. High-Performance Lead-Free Piezoceramics with High Curie Temperatures. Adv Mater. 2015; 27(43): 6976–6982. https://doi.org/10.1002/adma.201502424

20. Wang K, Yao FZ, Jo W, Gobeljic D, Shvartsman VV, Lupascu DC, et al. Temperature-insensitive (K,Na)NbO3-based lead-free piezoactuator ceramics. Adv Funct Mater. 2013; 23(33): 4079–4086. https://doi.org/10.1002/adfm.201203754

21. Fu J, Zuo R, Wang X, Li L. Polymorphic phase transition and enhanced piezoelectric properties of LiTaO3-modified (Na0.52K0.48) (Nb 0.93Sb0.07)O3 lead-free ceramics. *J Phys D Appl Phys*. 2009; 42(1): 012006. https://doi.org/10.1088/0022-3727/42/1/012006
22. Zhao C, Wang H, Xiong J, Wu J. Composition-driven phase boundary and electrical properties in (Ba0.94Ca0.06)(Ti1-xMx)O3 (M = Sn, Hf, Zr) lead-free ceramics. Dalt Trans. 2016; 45(15): 6466–6480. https://doi.org/10.1039/c5dt04891e
